# Integration of Continuous-Time Dynamics in a Spiking Neural Network Simulator

**DOI:** 10.3389/fninf.2017.00034

**Published:** 2017-05-24

**Authors:** Jan Hahne, David Dahmen, Jannis Schuecker, Andreas Frommer, Matthias Bolten, Moritz Helias, Markus Diesmann

**Affiliations:** ^1^School of Mathematics and Natural Sciences, Bergische Universität WuppertalWuppertal, Germany; ^2^Institute of Neuroscience and Medicine (INM-6), Institute for Advanced Simulation (IAS-6), JARA BRAIN Institute I, Jülich Research CentreJülich, Germany; ^3^Department of Physics, Faculty 1, RWTH Aachen UniversityAachen, Germany; ^4^Department of Psychiatry, Psychotherapy and Psychosomatics, Medical Faculty, RWTH Aachen UniversityAachen, Germany

**Keywords:** rate models, spiking neural network simulator, stochastic (delay) differential equations, waveform relaxation, parallelization, supercomputing

## Abstract

Contemporary modeling approaches to the dynamics of neural networks include two important classes of models: biologically grounded spiking neuron models and functionally inspired rate-based units. We present a unified simulation framework that supports the combination of the two for multi-scale modeling, enables the quantitative validation of mean-field approaches by spiking network simulations, and provides an increase in reliability by usage of the same simulation code and the same network model specifications for both model classes. While most spiking simulations rely on the communication of discrete events, rate models require time-continuous interactions between neurons. Exploiting the conceptual similarity to the inclusion of gap junctions in spiking network simulations, we arrive at a reference implementation of instantaneous and delayed interactions between rate-based models in a spiking network simulator. The separation of rate dynamics from the general connection and communication infrastructure ensures flexibility of the framework. In addition to the standard implementation we present an iterative approach based on waveform-relaxation techniques to reduce communication and increase performance for large-scale simulations of rate-based models with instantaneous interactions. Finally we demonstrate the broad applicability of the framework by considering various examples from the literature, ranging from random networks to neural-field models. The study provides the prerequisite for interactions between rate-based and spiking models in a joint simulation.

## 1. Introduction

Over the past decades, multiple strategies of neural network modeling have emerged in computational neuroscience. Functionally inspired top-down approaches that aim to understand computation in neural networks typically describe neurons or neuronal populations in terms of continuous variables, e.g., firing rates (Hertz et al., 1991; Schöner et al., 2015). Rate-based models originate from the seminal works by Wilson and Cowan (1972) and Amari (1977) and were introduced as a coarse-grained description of the overall activity of large-scale neuronal networks. Being amenable to mathematical analysis and exhibiting rich dynamics such as multistability, oscillations, traveling waves, and spatial patterns (see e.g., Roxin et al., 2005), rate-based models have fostered progress in the understanding of memory, sensory and motor processes including visuospatial working memory, decision making, perceptual rivalry, geometric visual hallucination patterns, ocular dominance and orientation selectivity, spatial navigation, and movement preparation (reviewed in Coombes, 2005; Bressloff, 2012; Kilpatrick, 2015). On the brain scale, rate models have been used to study resting-state activity (Deco et al., 2011) and hierarchies of time scales (Chaudhuri et al., 2015). Ideas from functional network models have further inspired the field of artificial neuronal networks in the domain of engineering (Haykin, 2009).

In contrast, bottom-up approaches are motivated by the microscopic dynamics of individual neurons. Biophysically grounded spiking neuron models that simulate the time points of action potentials can explain a variety of salient features of microscopic neural activity observed *in vivo*, such as spike-train irregularity (Softky and Koch, 1993; van Vreeswijk and Sompolinsky, 1996; Amit and Brunel, 1997; Shadlen and Newsome, 1998), membrane-potential fluctuations (Destexhe and Paré, 1999), asynchronous firing (Brunel, 2000; Ecker et al., 2010; Renart et al., 2010; Ostojic, 2014), correlations in neural activity (Gentet et al., 2010; Okun and Lampl, 2008; Helias et al., 2013), self-sustained activity (Ohbayashi et al., 2003; Kriener et al., 2014), rate distributions across neurons (Griffith and Horn, 1966; Koch and Fuster, 1989; Roxin et al., 2011) and across laminar populations (Potjans and Diesmann, 2014), as well as resting state activity (Deco and Jirsa, 2012). Furthermore, in population-density approaches, statistical descriptions of neuronal populations neglect the identities of individual neurons and describe the dynamics of homogeneous populations in terms of probability densities (reviewed e.g., Deco et al., 2008). These approaches capture the time-dependent population activity enabling the investigation of phenomena like desynchronization (de Kamps, 2013) and computational properties of cortical circuits (Cain et al., 2016).

Simulation of rate-based models goes back to the works by Grossberg (1973), McClelland and Rumelhart (1981), Feldman and Ballard (1982), and the PDP group (Rumelhart et al., 1986). Various specialized tools have developed since then (O'Reilly, 2014), such as *PDP*++ (McClelland and Rumelhart, 1989; O'Reilly et al., 2000), the *Neural Simulation Language* (Weitzenfeld et al., 2002), *emergent* (O'Reilly et al., 2012), the simulation platform *DANA* (Rougier and Fix, 2012), *TheVirtualBrain* (Sanz Leon et al., 2013), *Topographica* (Bednar, 2009) and the *Neural Field Simulator* (Nichols and Hutt, 2015). Similarly, efficient simulators for population-density approaches (*MIIND*: de Kamps et al., 2008, *DiPDE*: Cain et al., 2016) as well as spiking neural networks (see Brette et al., 2007 for a review) have evolved. The foci of the latter range from detailed neuron morphology (*NEURON*: Carnevale and Hines, 2006, *GENESIS*: Bower and Beeman, 2007) to an abstraction of neurons without spatial extent (*NEST:* Bos et al., 2015, *BRIAN*: Goodman and Brette, 2013). Such open-source software, combined with interfaces and simulator-independent languages (Davison et al., 2008; Djurfeldt et al., 2010, 2014), supports maintainability, reproducibility, and exchangeability of models and code, as well as community driven development. However, these tools are restricted to either rate-based or spike-based models only.

The situation underlines that bottom-up and top-down strategies are still mostly disjoint and a major challenge in neuroscience is to form a bridge between the spike- and rate-based models (Abbott et al., 2016), and, more generally, between the fields of computational neuroscience and cognitive science. From a practical point of view, a common simulation framework would allow the exchange and the combination of concepts and code between the two descriptions and trigger interaction between the corresponding communities. This is in particular important since recent advances in simulation (Djurfeldt et al., 2008; Hines et al., 2008; Kumar et al., 2010; Hines et al., 2011; Helias et al., 2012; Kunkel et al., 2014) and computing technology (Jülich Supercomputing Centre, 2015; Miyazaki et al., 2012) enable full-density bottom-up models of complete circuits (Potjans and Diesmann, 2014; Markram et al., 2015). In particular, it has become feasible to build spiking models (Schmidt et al., 2016) that describe the same macroscopic system as rate-based descriptions (Chaudhuri et al., 2015).

The relation between the different model classes is one focus of theoretical neuroscience. Assuming homogeneity across neurons, population-density methods reformulate the spiking dynamics as a dynamical equation for the probability density that captures the time evolution of the population activity (Knight, 1972; Gerstner, 1995, 2000). Under certain assumptions allowing the neglect of fluctuations in the input to neurons, a set of coupled differential equations for the population-averaged firing rate and membrane potential can be derived (Montbrió et al., 2015). For asynchronous irregular activity, input fluctuations can be taken into account in a diffusion approximation which leads to Fokker-Planck mean-field theory that can be used to determine homogeneous stationary state activities of spiking networks (Siegert, 1951; Brunel, 2000). The Fokker-Planck ansatz is, however, not limited to the population level, but can yield an heterogeneous stationary state firing rate across individual neurons in the network (Sadeh and Rotter, 2015). The dynamics of rate fluctuations around the background activity can be obtained using linear response theory on the population level (Brunel and Hakim, 1999) or the level of individual neurons (Lindner et al., 2005; Ostojic and Brunel, 2011; Trousdale et al., 2012; Grytskyy et al., 2013; Schuecker et al., 2015) yielding effective rate models on the population or single-neuron level. An alternative to linear response theory is given by moment expansions for mode decompositions of the Fokker-Planck operator (Mattia and Del Guidice, 2002, 2004; Deco et al., 2008).

An alternative derivation of rate-based dynamics aims at a closure of equations for synaptic currents of spiking networks in a coarse-graining limit by replacing spiking input with the instantaneous firing rate (Bressloff, 2012). Using field-theoretical methods (Buice and Chow, 2013) that were originally developed for Markovian network dynamics (Buice and Cowan, 2007; Buice et al., 2010) allows a generalization of this approach to fluctuations in the input (Bressloff, 2015).

In any case, the cascade of simplifications from the original spiking network to the rate-based model involves a combination of approximations which are routinely benchmarked in comparative simulations of the two models. A unified code base that features both models would highly simplify these validations rendering duplication of code obsolete.

In many cases rate models represent populations of spiking neurons. Thus, a hybrid model, employing both types of models in a multi-scale modeling approach, would contain a relatively large number of spiking neurons compared to the number of rate units. Despite the large size of the spiking network, the dynamics still features finite-size fluctuations (Ginzburg and Sompolinsky, 1994; Meyer and van Vreeswijk, 2002; Mattia and Del Guidice, 2004; Helias et al., 2013; Schwalger et al., 2016), and a downscaling of the network can generally not be performed without changing correlations (van Albada et al., 2015). Thus, it is crucial that a common simulation framework is able to handle real-sized spiking networks. In addition, the employed mean-field theories exploit the large number of neurons in biological networks. In fact, they are strictly valid only in the thermodynamic limit *N* → ∞ (Helias et al., 2014). Therefore, in the above mentioned validation studies, the spiking networks are typically large. Thus, a common simulation framework should be optimized for spiking neurons rather than rate-based models.

Current spiking network simulators solve the neuronal dynamics in a distributed and parallel manner. They exploit the point-event like nature of the spike interaction between neurons, for example in event-based simulation schemes. Here, the term event-based denotes the update scheme of synapses. In contrast, for the neuron dynamics a globally time-driven update scheme is more beneficial due to the large total number of incoming events per neuron (Morrison et al., 2005). Moreover, a purely event-driven scheme cannot be efficiently distributed since it requires a central event queue (Hanuschkin et al., 2010). Spiking point-neuron models furthermore interact in a delayed fashion. The delays mimic the synaptic transmission and the propagation times along axons and dendrites. For the duration of the minimal delay *d*_min_ in a network, the dynamics of all neurons is decoupled. Hence, during *d*_min_, the neurons can be updated independently without requiring information from other neurons. Distributed processes therefore need to communicate spikes only after this period (Morrison et al., 2005). Due to considerable latencies associated with each communication, this scheme significantly improves performance and scalability of current simulators. Although rate-based models require communication of continuous state variables, the *d*_min_-communication scheme can be used if these interactions have a delay. However, many rate based-models consider instantaneous interactions between units (see Bressloff, 2012, and references therein), typically for analytical convenience in quasi-static situations where delays do not matter. A priori, these interactions require communication between units at each time step.

The present study provides the concepts and a reference implementation for the embedding of continuous-time dynamics in a spiking network simulator. To exploit existing functionality we choose as a platform the open source simulation code NEST (Gewaltig and Diesmann, 2007; Bos et al., 2015) which is a scalable software used on machines ranging from laptops to supercomputers. The software is used by a considerable user community and equipped with a Python interface, supports the construction of complex networks, and shields the neuroscientist from the difficulties of handling a model description, potentially including stochastic components, in a distributed setting (Morrison et al., 2005; Plesser et al., 2015). Within this framework we introduce an iterative numerical solution scheme that reduces communication between compute nodes. The scheme builds on the waveform-relaxation technique (Lelarasmee, 1982) already employed for gap-junction interactions (Hahne et al., 2015).

Our study begins with a brief review of numerical solution schemes for ordinary and stochastic (delay) differential equations in Section 2 and their application to neural networks in Section 2.2. Subsequently, we develop the concepts for embedding rate-based network models into a simulation code for spiking networks, adapt the waveform-relaxation scheme, and detail an extendable implementation framework for rate models in terms of templates (Section 2.3). In Section 3, different numerical schemes are evaluated as well as the scalability of our reference implementation. We illustrate the applicability of the framework to a broad class of network models on the examples of a linear network model (Grytskyy et al., 2013), a nonlinear network model (Sompolinsky et al., 1988; Goedeke et al., 2016), a neural field model (Roxin et al., 2005), and a mean-field description (Wong and Wang, 2006) of the stationary activity in a model of the cortical microcircuit (Potjans and Diesmann, 2014; Schuecker et al., 2017). Straight-forward generalizations are briefly mentioned at the end of the Results section, before the work concludes with the Discussion in Section 4. The technology described in the present article will be made available with one of the next major releases of the simulation software NEST as open source. The conceptual and algorithmic work is a module in our long-term collaborative project to provide the technology for neural systems simulations (Gewaltig and Diesmann, 2007).

## 2. Materials and methods

Rate-based single neuron and population models are described in terms of differential equations that often include delays and stochastic elements. Before we turn to the implementation of such models in computer code (Section 2.3) we review how such systems are mathematically solved and in particular how the stochastic elements are commonly interpreted with the aim to avoid an *ad-hoc* design. A stochastic differential equation (SDE) is defined by the corresponding stochastic integral equation. Let *W*(*t*) denote a Wiener process, also called Standard Brownian motion. For the initial condition *X*(*t*_0_) = *X*_0_ an Itô-SDE in its most general form satisfies

(1) $$X(t)=X_{0}+\int_{t_{0}}^{t}a(s,X(s))ds+\int_{t_{0}}^{t}b(s,X(s))dW(s),$$

where the second integral is an Itô integral

$$\int_{t_{0}}^{t}Y(s)dW(s)\;:\text{​​}=\underset{n\to \infty }{\lim}\sum_{i=1}^{n}Y_{i-1}\cdot (W_{i}-W_{i-1})$$

with $Y_{i}=Y\left(t_{0}+i\cdot \frac{t-t_{0}}{n}\right)$ and $W_{i}=W\left(t_{0}+i\cdot \frac{t-t_{0}}{n}\right)$. Alternatively, the second integral can be chosen as a Stratonovich integral, indicated by the symbol ◦,

$$\int_{t_{0}}^{t}Y(s)◦dW(s)\;:\text{​​}=\underset{n\to \infty }{\lim}\sum_{i=1}^{n}\frac{Y_{i-1}+Y_{i}}{2}(W_{i}-W_{i-1})$$

which approximates *Y*(*s*) with the mid-point rule. In this case, the corresponding SDE is called a Stratonovich-SDE. We refer to Kloeden and Platen (1992) and Gardiner (2004) for a derivation and a deeper discussion on the differences between the two types of stochastic integrals. In the case of additive noise (*b*(*t, X*(*t*)) = *b*(*t*)) the Itô and Stratonovich integrals coincide. If furthermore the noise is constant (*b*(*t, X*(*t*)) = σ = const.) the integrals can be solved analytically

$$\begin{array}{l} \int_{t_{0}}^{t}\sigma \;dW(s)=\int_{t_{0}}^{t}\sigma ◦dW(s)=\underset{n\to \infty }{\lim}\sigma \cdot \sum_{i=1}^{n}\left(W_{i}-W_{i-1}\right)\\ \;=\sigma \cdot (W(t)-W(t_{0})) \end{array}$$

with $W\left(t\right)-W\left(t_{0}\right)\sim N\left(0,t-t_{0}\right)$. In the following, we focus on Itô-SDEs only.

The differential notation corresponding to Equation (1) reads

(2) dX(t)=a(t,X(t))dt+b(t,X(t))dW(t)

and denotes an informal way of expressing the integral equation. Another widely used differential notation, called the Langevin form of the SDE, is mostly employed in physics. It reads

(3) $$\frac{dX(t)}{dt}=a(t,X(t))+b(t,X(t))\xi (t),$$

where ξ(*t*) is a Gaussian white noise with 〈ξ(*t*)〉 = 0 and 〈ξ(*t*)ξ(*t*′)〉 = δ(*t* − *t*′). Using the Fokker-Planck equation one obtains

$$\int_{0}^{t}\xi (t^{\prime })dt^{\prime }=W(t),$$

which is a paradox, as one can also show that *W*(*t*) is not differentiable (Gardiner, 2004, Chapter 4). Mathematically speaking this means that Equation (3) is not strictly well-defined. The corresponding stochastic integral equation

$$X(t)=X_{0}+\int_{t_{0}}^{t}a(s,X(s))ds+\int_{t_{0}}^{t}b(s,X(s))\xi (s)ds,$$

however, can be interpreted consistently with Equation (1) as *dW*(*t*) ≡ ξ(*t*)*dt*.

### 2.1. Approximate numerical solution of SDEs

Similar to ordinary differential equations most stochastic differential equations cannot be solved analytically. Neuroscience therefore relies on approximate numerical schemes to obtain the solution of a given SDE. This section presents some basic numerical methods. Let Δ*t* denote the fixed step size, *t*_*k*_ = *t*_0_ + *k*Δ*t* the grid points of the discretization for *k* = 0, …, *n*, and *X*_*k*_ the approximation for *X*(*t*_*k*_) obtained by the numerical method, at which *X*_0_ is the given initial value. We consider systems of *N* stochastic differential equations:

(4) dX(t)=a(t,X(t))dt+b(t,X(t))dW(t)

with initial condition *X*(*t*_0_) = *X*_0_. Here, *X*(*t*) = (*X*^1^(*t*), …, *X*^*N*^(*t*)) and *W*(*t*) = (*W*^1^(*t*), …, *W*^*N*^(*t*)) denote *N*-dimensional vectors and *a*:ℝ^*N*^ → ℝ^*N*^ and *b*:ℝ^*N*^ → ℝ^*N*^ are *N*-dimensional functions. *W*(*t*) is an *N*-dimensional Wiener process, i.e., the components *W*^*i*^(*t*) are independent and identically distributed.

#### 2.1.1. Euler-maruyama

The Euler-Maruyama method is a generalization of the forward Euler method for ordinary differential equations (ODE). Accordingly, it approximates the integrands in Equation (1) with their left-sided values. The update formula reads

(5) $$X_{k+1}=X_{k}+a(t_{k},X_{k})\cdot \Delta t+b(t_{k},X_{k})\cdot \Delta W_{k}$$

with $\Delta W_{k}=W\left(t_{k+1}\right)-W\left(t_{k}\right)\sim N\left(0,\Delta t\right)$ for *k* = 0, …, *n* − 1.

#### 2.1.2. Semi-implicit euler

The (semi-)implicit Euler method is a generalization of the backwards Euler method for ODEs. The update formula reads

(6) $$X_{k+1}=X_{k}+a(t_{k+1},X_{k+1})\cdot \Delta t+b(t_{k},X_{k})\cdot \Delta W_{k}.$$

The resulting scheme requires the solution of a system of nonlinear algebraic equations. Standard techniques for the solution of the system are Newton iteration and fixed-point iteration (Kelley, 1995). The method is sometimes called semi-implicit, because the function *b* is still evaluated at (*t*_*k*_, *X*_*k*_) instead of (*t*_*k*+1_, *X*_*k*+1_). However, a fully implicit Euler scheme for SDEs is not practicable (see Kloeden and Platen, 1992, Chapter 9.8) and thus the term implicit Euler usually refers to the semi-implicit method and is used in the following.

#### 2.1.3. Exponential euler

The exponential Euler method relies on the assumption that *a*(*t, X*(*t*)) consists of a linear part and a nonlinear remainder, i.e.,

a(t,X(t))=A·X(t)+f(t,X(t))

with *A* ∈ ℝ^*N*×*N*^. The idea is to solve the linear part exactly and to approximate the integral of the nonlinear remainder and the Itô integral with an Euler-like approach. Variation of constants for Equation (4) yields

$$\begin{array}{l} X(t)=e^{A(t-t_{0})}X_{0}+\int_{t_{0}}^{t}e^{A(t-s)}f(s,X(s))ds\\ \;+\;\int_{t_{0}}^{t}e^{A(t-s)}b(s,X(s))dW(s). \end{array}$$

There are several versions of stochastic exponential Euler methods that differ in the approximation of the integrals. Unfortunately a standardized nomenclature to distinguish the methods is so far missing. The simplest approach, sometimes named stochastic Lawson-Euler scheme (e.g., Komori and Burrage, 2014), approximates the integrands with their left-sided values

$$X_{k+1}=e^{A\Delta t}X_{k}+e^{A\Delta t}f(t_{k},X_{k})\cdot \Delta t+e^{A\Delta t}b(t_{k},X_{k})\cdot \Delta W_{k}.$$

More advanced schemes approximate the nonlinear part by keeping *f*(*s, X*(*s*)) constant for [*t*_0_, *t*) and solving the remaining integral analytically

$$\begin{array}{l} \int_{t_{0}}^{t}e^{A(t-s)}f(s,X(s))ds\approx \int_{t_{0}}^{t}e^{A(t-s)}f(t_{0},X(t_{0}))ds\\ \;=\;A^{-1}(e^{A(t-t_{0})}-I)\cdot f(t_{0},X(t_{0})). \end{array}$$

Here *I* denotes the *N* × *N* identity matrix. The same technique can be used for the Itô integral

(7) $$\int_{t_{0}}^{t}e^{A(t-s)}b(s,X(s))dW(s)\approx \int_{t_{0}}^{t}e^{A(t-s)}b(t_{0},X(t_{0}))dW(s).$$

For a single SDE, Shoji (2011) proposed a method where the remaining integral $\int_{t_{0}}^{t}e^{a\left(t-s\right)}dW\left(s\right)$ with *a* ∈ ℝ is approximated by $\int_{t_{0}}^{t}\alpha dW\left(s\right)$, such that α ∈ ℝ is chosen to minimize the mean-square error. This results in a similar approximation as for the nonlinear part. Komori and Burrage (2014) adapted this approach for systems of SDEs. The scheme reads

$$\begin{array}{l} X_{k+1}=e^{A\Delta t}X_{k}+A^{-1}(e^{A\Delta t}-I)\cdot f(t_{k},X(t_{k}))\\ \;+\;\frac{1}{\Delta t}\cdot A^{-1}(e^{A\Delta t}-I)\cdot b(t_{k},X_{k})\cdot \Delta W_{k}. \end{array}$$

Alternatively, calculating the variance of *X*(*t*) within the approximation (7), amounts to (Adamu, 2011)

$$\begin{array}{l} \text{Var}\;(X(t))=b{\left(t_{0},X(t_{0})\right)}^{2}\cdot \text{Var}(\int_{t_{0}}^{t}e^{A(t-s)}dW(s))\\ \;=\;b{\left(t_{0},X(t_{0})\right)}^{2}\cdot A^{-1}(\frac{e^{2A(t-t_{0})}-I}{2}). \end{array}$$

The corresponding scheme reads

(8) $$\begin{array}{l} X_{k+1}=e^{A\Delta t}X_{k}\;+\;A^{-1}(e^{A\Delta t}-I)\cdot f(t_{k},X(t_{k}))\\ \;+\;\sqrt{A^{-1}\left(\frac{e^{2A\Delta t}-I}{2}\right)}\cdot b(t_{k},X_{k})\cdot \eta_{k} \end{array}$$

with $\eta_{k}\sim N\left(0,1\right)$ and yields the exact solution of the system if *a*(*t, X*(*t*)) = *A* · *X*(*t*) and *b*(*t, X*(*t*)) = const., since *X*(*t*) has Gaussian statistics in this case (Risken, 1996). Therefore, in the following we exclusively employ (Equation 8) and just refer to it as the stochastic exponential Euler scheme. For more detailed reviews on the different stochastic exponential Euler methods we refer to Adamu (2011) and Komori and Burrage (2014).

### 2.2. Network of rate models

We now consider networks of *N* rate-based units where each unit receives recurrent input from the network. The system fulfills the Itô-SDEs

(9) $$\begin{array}{l} \tau^{i}dX^{i}(t)=\left[-X^{i}(t)\;+\;\mu^{i}\;+\;\phi \left(\sum_{j=1}^{N}w^{ij}\psi \left(X^{j}(t-d^{ij})\right)\right)\right]dt\\ \;+\;\sqrt{\tau^{i}}\sigma^{i}dW^{i}(t)\;i=1,\ldots ,N \end{array}$$

with possibly nonlinear input-functions ϕ(*x*) and ψ(*x*), connection weights *w*^*ij*^, mean input μ^*i*^, and optional delays *d*^*ij*^ ≥ 0. The corresponding Fokker-Planck equation shows that the parameter σ^*i*^ ≥ 0 controls the variance of *X*^*i*^(*t*) and the time constant τ^*i*^ > 0 its temporal evolution. For readability, from here on we omit unit indices for σ, τ, μ, and *d*. The considered class of rate models only contains additive noise. Therefore, as noted above, the system (Equation 9) can be written as Stratonovich-SDEs without the need for change in the employed numerical methods. For an illustrative purpose we explicitly state the different explicit solution schemes for the network dynamics (Equation 9) with *d* = 0. The Euler-Maruyama update step reads

(10) $$\begin{array}{l} X_{k+1}^{i}=X_{k}^{i}+\left[-X_{k}^{i}+\mu +\phi \left(\sum_{j=1}^{N}w^{ij}\psi \left(X_{k}^{j}\right)\right)\right]\frac{1}{\tau }\Delta t\\ \;+\;\frac{1}{\sqrt{\tau }}\sigma \Delta W_{k}^{i}\;. \end{array}$$

The implicit Euler update formula evaluates *X* at *k* + 1 instead of *k* within the square brackets. This turns Equation 10 into a system of nonlinear algebraic equations for which the fixed-point iteration

(11) $$X_{k+1}^{i,m+1}=\Phi (X_{k+1}^{1,m},\ldots ,X_{k+1}^{N,m})$$

with initial value $X_{k+1}^{i,0}=X_{k}^{i}$ and the choice of

(12) $$\Phi =\frac{X_{k}^{i}+\left[\mu +\phi \left(\sum_{j=1}^{N}w^{ij}\psi \left(X_{k+1}^{j,m}\right)\right)\right]\frac{1}{\tau }\Delta t+\frac{1}{\sqrt{\tau }}\sigma \Delta W_{k}^{i}}{1+\Delta t/\tau }\;$$

yields the solution.

For nonlinear ϕ(*x*) or ψ(*x*) the exponential Euler update step is

(13) $$\begin{array}{l} X_{k+1}^{i}=e^{-\Delta t/\tau }X_{k}^{i}\;+\;\left(1-e^{-\Delta t/\tau }\right)\left[\mu +\phi \left(\sum_{j=1}^{N}w^{ij}\psi \left(X_{k}^{j}\right)\right)\right]\\ \;+\;\sqrt{\frac{1}{2}(1-e^{-2\Delta t/\tau })}\sigma \eta_{k}^{i} \end{array}$$

with $\eta_{k}^{i}\sim N\left(0,1\right)$. As *A* = −*I* is a diagonal matrix, the exponential Euler scheme does not rely on a matrix exponential, but decomposes into *N* equations with scalar exponential functions. Note that with a linear choice, ϕ(*x*) = ψ(*x*) = *x*, the system of SDEs can be written in matrix notation

(14) $$\tau dX(t)=\left[A\cdot X(t)+\mu \right]dt+\sqrt{\tau }\sigma dW(t)\;i=1,\ldots ,N$$

with *A* = −*I* + *W* and $W={\left(w^{ij}\right)}_{N\times N}$. Here the stochastic exponential Euler scheme (Equation 8) yields the exact solution of the system.

The numerical schemes presented in Section 2.1 are developed for SDEs (*d* = 0), but can analogously be used for stochastic delay differential equations (SDDEs) (*d* > 0), if the delay *d* is a multiple of the step size Δ*t*. For the calculation of the approximation $X_{k+1}^{i}$ in time step *k* + 1 the recurrent input is then evaluated from $\frac{d}{\Delta t}$ steps earlier, i.e., from $X_{k-\frac{d}{\Delta t}}^{j}$ for the explicit methods.

### 2.3. Implementation in spiking network simulation code

This section describes the embedding of rate-based models (Section 2.2) in a simulation code for spiking neuronal networks. The Appendix (Section A.2) illustrates how to create, connect and record activity from rate models in our reference implementation.

The software architecture for rate models is based on existing concepts: Morrison et al. (2005) describe distributed buffers for the storage of delayed interactions and the technique to consistently generate random numbers in a distributed setting, and Hahne et al. (2015) introduce so called secondary events, that allow the communication of continuous state variables, like membrane potentials or rates, between neurons or rate units respectively. Events provide an abstraction layer on top of the MPI communication which allows the implementation of neuron models without explicit reference to MPI calls. Unlike primary events which are used to transmit the occurrence of spikes at discrete points in time, secondary events occur on a regular time grid. These concepts are designed to be compatible with the parallel and distributed operation of a simulation kernel for spiking neuronal networks, ensuring an efficient use of clusters and supercomputers (Helias et al., 2012). This allows researchers to easily scale up network sizes to more realistic number of neurons. The highly parallelizable structure of modern simulation codes for spiking neuronal networks, however, also poses restrictions on the utilizable numerical methods.

#### 2.3.1. Parallelization and restrictions

Parallelization for spiking neuronal networks is achieved by distributing neurons over compute nodes. Since the dynamics of spiking neurons (in the absence of gap junctions) is decoupled for the duration of the minimal synaptic delay *d*_min_ of the connections in the network, the states of the neurons can be propagated independently for this time interval. Thus, it is sufficient to specify solvers on the single-neuron level. The spike times, i.e., the mediators of interaction between neurons, are then communicated in steps of *d*_min_.

As a result of this structure the global connectivity of the network is unknown to the single neuron. The neuron object sends and receives events handled by an object on the compute node harboring the neuron termed network manager. However, the network manager only knows the incoming connections of the neurons on the compute node.

This poses restrictions on the use of implicit schemes. It is impossible to employ the implicit Euler scheme (Equation 6) with Newton iteration, which would require the simultaneous solution of a system of nonlinear algebraic equations with information distributed over all compute nodes. The use of the implicit Euler scheme with fixed-point iteration is however compatible with this structure. To this end, the scheme (Equation 6) needs to be formulated as a fixed-point iteration on the single-unit level (see Section 2.2) and the updated rates need to be communicated to the connected units after every iteration until some convergence criterion is met. The convergence of the fixed-point iteration is however only guaranteed if the scheme Φ is contractive (see e.g., Kelley, 1995, their Section 4.2), which poses restrictions on the employed step size Δ*t*. Section 3.1 investigates if the implementation can gain stability or accuracy from using the implicit Euler method with fixed-point iteration and if the payoff is large enough to justify the additional effort of an iterative solution scheme.

The restricted knowledge of connectivity also limits the usage of the exponential Euler method. In the case of a linear rate model, we are unable to add the influence from all other rate units to the matrix *A* in Equation (14), because most of these connections are unknown at the single-unit level. Therefore, we use the exponential Euler method with *A* = −*I* resulting in the update formula (13). This also has the benefit of avoiding the need to numerically evaluate a general matrix exponential as *A* is a diagonal matrix (see Section 2.2 for details).

#### 2.3.2. Implementation

This section describes the additional data structure required for the implementation of rate-based models. While the naming convention refers to our reference implementation in the simulation software NEST, the employed algorithms and concepts are portable to other parallel spiking network simulators. As a result of the previous section and our analysis of the numerical schemes below (see Section 3.1) we restrict the discussion and our reference implementation to the exponential Euler method where we assume *A* = −*I* and identify Δ*t* = *h* with *h* denoting the global computation step size (Morrison et al., 2005). We have to distinguish the cases of connections with delay (*d* > 0) and connections without delay (*d* = 0). The former case is similar to spiking interaction: assuming a connection from unit *i* to unit *j*, the rate of unit *i* needs to be available at unit *j* after $\frac{d}{h}$ additional time steps. This can be ensured if the delay of the connection is considered in the calculation of the minimal delay *d*_min_ that determines the communication interval. After communication the rate values are stored in a ring buffer of unit *j* until they are due (Morrison and Diesmann, 2008). In the case of an instantaneous connection, the rate of unit *i* at time *t*_0_ needs to be known at time *t*_0_ at the process which updates unit *j* from *t*_0_ to *t*_0_ + *h*. Therefore, communication in every step is required for instantaneous rate connections, i.e., setting *d*_min_ = *h*.

Due to the conceptual differences between instantaneous and delayed interactions (for the conceptual difference in the case of spiking interaction see Morrison and Diesmann, 2008) we employ two different connection types (delay_rate_connection, rate_connection) and associated secondary events (DelayRateNeuronEvent, RateNeuronEvent). This subdivision simplifies the discrimination of connections on the single-unit level, while still allowing for simultaneous use of instantaneous and delayed connections in the same rate model.

The large diversity of rate models (Equation 9) imposes a challenge for code maintenance and efficiency: Each combination of nonlinearities ϕ(*x*) and ψ(*x*) constitutes its own model. All of these models can be implemented in the exact same way, except for the evaluation of the nonlinearities. A template class (rate_neuron_ipn) providing a base implementation for rate models of category (9) avoids code duplication. Nevertheless, we restrict the reference implementation to one nonlinearity per model. This keeps the zoo of rate models small and to our knowledge covers the majority of rate models.

The template rate model class is instantiated with an object that represents the nonlinearity. Being instantiated at compile time, this template solution does not incur additional overhead at run time compared to a solution using polymorphy (inheritance). A boolean class member linear_summation determines if the nonlinearity should be interpreted as ϕ(*x*) (true, default value) or ψ(*x*) (false). The respective other function is assumed to be the identity function. The boolean parameter is evaluated in every update step of each unit. Deciding upon the type of nonlinearity at compile time would improve efficiency. In the present architecture this would, however, result in twice as many template instances for a given set of gain functions. With the future capabilities of code generation (Plotnikov et al., 2016) in mind it might be beneficial to elevate the constant boolean member object to a constant template parameter to allow compilers efficient preprocessing and at the same time profit from the code reliability achievable by modern C++ syntax. The present base implementation reduces the effort of creating a specific rate model of category (9) to the specification of an instance of the template class. Afterwards an actual rate model can be instantiated in a single line of code.

Table 1 gives an overview of template-derived rate models of the reference implementation. These models serve as examples for customized rate models. Activity of rate units can be recorded using the multimeter and the recordable rate.

**Table 1:** **Template-derived rate-based models**.

**Gain model**	**ϕ(*x*) or ψ(*x*)**
lin_rate	*g* · *x* with *g* ∈ ℝ
tanh_rate	tanh(*g* · *x*) with *g* ∈ ℝ
thresholdlin_rate	*g* · (*x* − θ) · *H*(*x* − θ) with *g*, θ ∈ ℝ

*Gain functions of the rate-based models available in the NEST reference implementation. The name of a particular rate model is formed by <gain model>_ipn. The ending ipn indicates input noise, as the noise directly enters the r.h.s. of Equation (9). H denotes the Heaviside function*.

In addition to these template-derived models of category (9) the reference implementation also contains a rate model called siegert_neuron. This model, described by (30) in Section 3.3.4 is used for mean-field analysis of complex networks and constitutes a special case with respect to the recurrent input from the network. Firstly, it requires a numerically stable implementation of the Siegert formula (see Section A.1). Secondly, Equations (28) and (29) demonstrate that for this model the input rates are weighted by separate factors. Thus, for connections between instances of this model two different weights need to be specified and the rate model must be able to handle this anomaly. Therefore, the siegert_neuron does not derive from our base class rate_neuron_ipn, but constitutes an independent class. It comes with the connection type diffusion_connection providing the weight parameters. Section A.2 motivates the parameter names and shows the usage of the model in the reference implementation.

#### 2.3.3. Reduction of communication using waveform-relaxation techniques

Instantaneous connections between rate-based models require communication after every time step, thus in intervals of the global computation step size *h*. This requires setting *d*_min_ = *h* and impairs the performance and scalability, especially on supercomputers where communication is particularly expensive, because it is associated with a considerable latency. Therefore, for simulations with instantaneous connections we additionally study an iterative approach based on waveform-relaxation techniques that arrives, after convergence, at the same results as the standard approach, but allows us to use communication on a coarser time grid. Originally waveform-relaxation methods were developed (Lelarasmee, 1982) and investigated (see e.g., Miekkala and Nevanlinna, 1987) for ODEs. More recently they have also been analyzed for SDEs (Schurz and Schneider, 2005) and successfully applied to large systems of SDEs (Fan, 2013). With respect to simulations of rate-based models with instantaneous connections the basic idea is to solve the dynamics of the single units independently for the duration of *T* = α*h* with α ≥ 2 by treating the influence of the other units as known over this period of time. The solution of the original SDEs is determined by iteratively solving the decoupled SDEs:

(15) $$\begin{array}{l} dX^{1,m}=a(t,X^{1,m},Z^{1,m-1})dt+b(t,X^{1,m},Z^{1,m-1})dW,\\ \;\vdots \\ dX^{i,m}=a(t,X^{i,m},Z^{i,m-1})dt+b(t,X^{i,m},Z^{i,m-1})dW,\\ \;\vdots \\ dX^{N,m}=a(t,X^{N,m},Z^{N,m-1})dt+b(t,X^{N,m},Z^{N,m-1})dW, \end{array}$$

where in the *m*-th iteration *Z*^*i*^ = (*X*^1^, …, *X*^*i* − 1^, *X*^*i*+1^, …, *X*^*N*^) is based on the solution of the *m* − 1-th iteration and hence acts as a given input to the *i*-th system. The solutions improve step by step with each iteration. Schurz and Schneider (2005) demonstrate the convergence of the method for SDEs under mild assumptions on *X*, *a* and *b*. For our specific application, systems of rate-based models with *b* = const., the influence of the stochastic part on the convergence is even less critical. Based on our previous results in the neuroscience context (Hahne et al., 2015), the method converges within only a couple of iterations, due to the weak coupling of the SDEs. For more details on waveform-relaxation methods and their application in the neuroscience context we refer to Hahne et al. (2015).

As outlined above, in a simulator for spiking neuronal networks the minimal delay *d*_min_ in the network defines the communication interval. By employing the waveform-relaxation method with *T* = *d*_min_ we retain this communication interval for simulations with instantaneous rate connections. To control the iteration interval *T* of the waveform-relaxation method, instantaneous connections contribute to the calculation of the minimal delay with an arbitrary user specified value given by the parameter wfr_comm_interval (see Table 2). Consequently the actual communication interval for waveform relaxation then is *T* = min (*d*_min_, wfr_comm_interval).

**Table 2:** **Parameters of the waveform-relaxation algorithm**.

**Parameter name**	**Type**	**Default**	**Description**
use_wfr	bool	true	Boolean parameter to enable (true) or disable (false) the use of the waveform-relaxation technique. If disabled and any rate-based units (or neurons supporting gap junctions) are present, communication in every step is automatically activated (*d*_min_ = *h*).
wfr_comm_interval	double	1.0 ms	Instantaneous rate connections (and gap junctions) contribute to the calculation of the minimal network delay with min (*d*_min_, wfr_comm_interval). This way the length of the iteration interval of the waveform relaxation can be regulated.
wfr_tol	double	10^−4^	Convergence criterion for waveform relaxation. The iteration is stopped if the rates of all units change less than wfr_tol from one iteration to the next.
wfr_max_iterations	int	15	Maximum number of iterations performed in one application of the waveform relaxation. If the maximum number of iterations has been carried out without reaching the accuracy goal the algorithm advances system time and the reference implementation issues a warning. Additional speed-up in the simulation of rate-based units can only be achieved by wfr_max_iterations < *d*_min_/*h*.
wfr_interpolation_order	int	3	This parameter is exclusively used for gap junctions (see Hahne et al., 2015, their Section 2.1.2) and has no influence on the simulation of rate-based models.

*The different parameters of the waveform-relaxation algorithm together with their C++ data-type, default value, and a brief description*.

**Figure 1:**
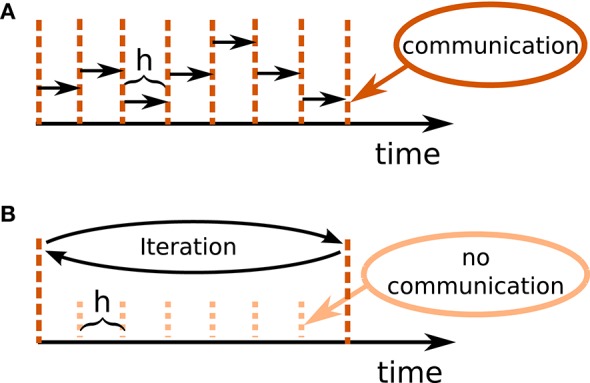
**Different communication strategies for distributed simulations**. Distance between neighboring dotted orange lines indicates computation time step of size *h*. Distance between neighboring dashed red lines symbolizes one communication interval where rates (and other events like spike events) are communicated at the end of the interval. **(A)** Standard solution for rate-based models: rates are communicated in every time step. **(B)** Iterative approach using waveform relaxation: rates are communicated only after $\frac{T}{h}$ steps and the entire interval is solved repeatedly.

Figure 1B illustrates the concept of the iterative approach in contrast to the standard procedure in panel A. The iterative approach requires the repeated solution of all time steps in the communication interval and converges to the solution obtained with the standard approach (Figure 1A). The iteration terminates when a user chosen convergence tolerance wfr_tol (see Table 2) is met. If the method needs less than *T*/*h* iterations, the approach reduces the overall number of communications required to obtain the solution. In conclusion, the avoidance of communication in every step comes for the price of additional computational load.

The coupling of neurons via gap junctions is instantaneous and continuous in time and thus constitutes a very similar problem to the rate dynamics. In order to combine gap junctions with spiking dynamics Hahne et al. (2015) already devised an iterative technique based on waveform-relaxation techniques and described a suitable framework. This framework can also be employed for the simulation of rate-based models with instantaneous connections. The dynamics of a neuron model supporting gap junctions is solved with an adaptive step-size ODE-solver, routinely carrying out several steps of the employed numerical method within one global computation time step *h*. The communication of a cubic interpolation of the membrane potential provides the solver with additional information, resulting in a more accurate solution than the one obtained from the standard approach. For rate-based models this additional benefit cannot be gained: The combination of an iterative method with an adaptive step-size solver is not applicable to SDEs, where the noise in each time step constitutes a random number. However, an iterative approach with fixed step size Δ*t* = *h* is applicable, as long as we ensure that the random noise applied to the units remains the same in every iteration. In Section 3.2 we investigate the performance of the iterative (Figure 1B) and the standard approach (Figure 1A) with a focus on large network simulations on supercomputers. In our reference implementation waveform relaxation can be enabled or disabled by a parameter use_wfr. Note that in the traditional communication scheme for spiking neuronal networks (Morrison et al., 2005) the first communication occurs earliest at the end of the first update step. Therefore, in the absence of waveform relaxation, the initial input to units from the network is omitted.

Table 2 summarizes the parameters of our reference implementation of the waveform-relaxation technique. A subset (wfr_interpolation_order, wfr_max_iterations, wfr_tol) was previously introduced by Hahne et al. (2015), but we rename them here to arrive at more descriptive names. The remaining parameters (use_wfr, wfr_comm_interval) result from the generalization to rate-based models.

## 3. Results

In the following, we assess the accuracy and stability of the different numerical solution schemes and benchmark the performance of the reference implementation on large-scale machines, with special focus on scalability and the comparison between the standard solution and the iterative approach using waveform relaxation for simulations with instantaneous connections. The iterative approach is only discussed with respect to efficiency, as the iteration always converges against the results of the standard approach within only a couple of iterations (for details see Section 2.3.3). The remainder of the section illustrates the application of the simulation framework to a selection of prominent problems in the neuroscientific literature.

### 3.1. Stability and accuracy of integration methods

In this section we investigate numerical methods (see Section 2.1) for the solution of SDEs that can be employed to solve the dynamics of rate-based units. We analyze the accuracy and stability of the different numerical methods to choose the best-suited method for application in a distributed simulation scheme of a spiking network simulation code. The analysis only covers methods compatible with a spiking neural network simulator, namely (i) the Euler-Maruyama method, (ii) the implicit Euler method solved with a parallelizable fixed-point iteration, and (iii) the exponential Euler method where the linear part is restricted to −*I*, in the following called scalar exponential Euler. The distributed representation of the global connectivity of the network rules out both the regular exponential Euler method with a nondiagonal matrix *A* and the implicit Euler method solved with Newton iteration (see Section 2.3.1 for details).

Consider an exactly solvable network of *N* linear rate units with μ = 0 (see also Section 2.2):

(16) $$\tau dX(t)=A\cdot X(t)dt+\sqrt{\tau }\sigma \;dW(t).$$

The exact solution of this system of SDEs coincides with the regular exponential Euler scheme and involves a matrix exponential and a matrix square root (Equation 8). We analyze two test cases, i.e., two different choices of *A*, to demonstrate different stability constraints. First, an all-to-all connected network with inhibitory connections of weight $w^{ij}=\frac{-1}{\sqrt{N}}$ and hence $A=-I+\frac{-1}{\sqrt{N}}\cdot 𝟙$, with 𝟙 denoting a *N* × *N* all-ones matrix (Cichocki et al., 2009). Second, a sparse balanced excitatory-inhibitory network where the number of excitatory units is four times larger than the number of inhibitory units. In this network, each unit receives input from a fixed number of 0.8 · *p* · *N* excitatory and 0.2 · *p* · *N* inhibitory randomly chosen source units with connection probability *p* and connection weights $\frac{1}{\sqrt{N}}$ and $\frac{-4}{\sqrt{N}}$, respectively. In the following we refer to the test cases as inhibitory all-to-all and sparse balanced e/i test case.

First we turn to the accuracy analysis. Although the exact solution of Equation (16) cannot be obtained with a distributed representation of *A* we can compute it using methods for numerical matrix computations implemented in MATLAB or Python (both provide an implementation of the same state-of-the-art algorithms, see Al-Mohy and Higham, 2009; Deadman et al., 2012). This way we obtain the exact solution within the accuracy of floating point numerics. This is the reference for computing the root mean square error (*RMSE*) of the different approximate methods. To employ the root mean square error in the context of stochastic differential equations we determine the reference solution for every tested step size and use the same random numbers for both, the reference solution and the approximative schemes.

**Figure 2:**
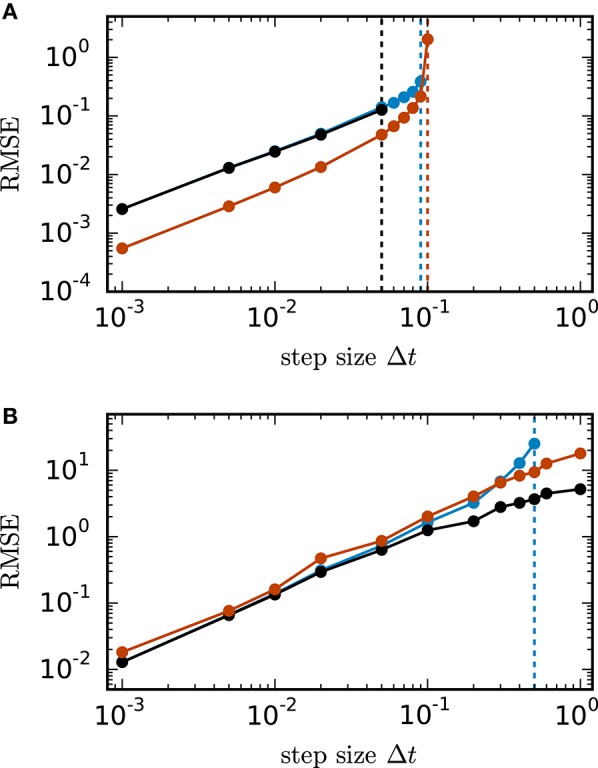
**Accuracy of numerical methods for two networks of linear rate units**. $RMSE=\sqrt{\frac{1}{N\left(t_{n}-t_{0}\right)}\overset{N}{\underset{i=1}{\sum }}\overset{n}{\underset{j=1}{\sum }}{\left(X_{j}^{i}-{\hat{X}}_{j}^{i}\right)}^{2}}$ of the solution *X* obtained by the approximate solvers (blue curve: Euler-Maruyama method, black curve: implicit Euler method solved with fixed-point iteration, red curve: scalar exponential Euler method) with respect to the reference solution $\hat{X}$ as a function of step size in double logarithmic representation. The respectively colored vertical lines mark the largest tested step size for which the corresponding methods deliver a solution with *RMSE* ≤ 10^10^. *RMSE* computed over 200.0 ms of biological time. **(A)** Inhibitory all-to-all test case. Network parameters: *N* = 400, μ = 0, σ = 10 and τ = 1 ms. **(B)** Sparse balanced e/i test case. Network parameters: *N* = 400, *p* = 0.2, μ = 0, σ = 10 and τ = 0.5 ms.

Figure 2 shows the root mean square error of the different numerical schemes for the two test cases with *N* = 400 units. In both test cases all investigated methods with decreasing step size converge toward the exact solution with convergence order 1, which is consistent with the established theory for SDEs with additive noise (Kloeden and Platen, 1992). Figure 2A shows the results for the inhibitory all-to-all test case. Here all three methods require a step size Δ*t* ≤ 0.1 to deliver reliable results. The implicit Euler scheme solved with fixed-point iteration even requires a step size Δ*t* ≤ 0.05. Within the stable region Δ*t* ≤ 0.1 the scalar exponential Euler yields more accurate results than the two other methods. Figure 2B shows the results for the sparse balanced e/i test case. Here all three methods achieve almost identical accuracy for Δ*t* ≤ 0.1 and stability problems only occur for the Euler-Maruyama method for step sizes Δ*t* > 0.5. For step sizes Δ*t* > 0.1 the implicit Euler method is more accurate than the other two methods.

To understand the stability issues shown in Figure 2 we turn to stability analysis. In the following we assume that *A* is diagonalizable, i.e., *A* = *T*^−1^*DT* with $T={\left(t^{ij}\right)}_{N\times N}\in {\mathbb{C}}^{N\times N}$ and *D* = diag(λ_1_, …, λ_*N*_), and transform the system of SDEs with *Z*(*t*) = *TX*(*t*). It follows

$$\tau dZ(t)=D\cdot Z(t)dt+\sqrt{\tau }\sigma TdW(t)$$

and *Z*(*t*_0_) = *TX*_0_. The transformed system consists of *N* equations of the form

(17) $$\tau dZ^{i}(t)=\lambda_{i}\cdot Z^{i}(t)dt+\sum_{j=1}^{N}\sqrt{\tau }\sigma t^{ij}dW^{j}(t)\;i=1,\ldots ,n$$

that depend on the eigenvalues λ_*i*_ of *A* and are pairwise independent except for the common contributions of the Wiener processes *W*^*j*^(*t*). In the following we only consider networks with bounded activity which requires eigenvalues λ_*i*_ ∈ ℂ with negative real part Re(λ_*i*_) < 0. The solution of the *i*-th transformed equation then satisfies

(18) $$\left|Z^{i}(t)-{\tilde{Z}}^{i}(t)|=e^{\lambda_{i}(t-t_{0})/\tau }|Z_{0}^{i}-{\tilde{Z}}_{0}^{i}|<|Z_{0}^{i}-{\tilde{Z}}_{0}^{i}\right|$$

for two different initial values $Z_{0}^{i}$ and ${\tilde{Z}}_{0}^{i}$. It is a desirable stability criterion that a numerical method applied to Equation (16) conserves this property. This requirement is closely related to the concept of A-stability for SDEs (see Kloeden and Platen, 1992, Chapter 9.8) and A- respectively B-stability for ODEs (Hairer and Wanner, 1991). To derive stability conditions for the numerical schemes, we apply one update step (*t*_0_ to *t*_1_ = *t*_0_ + Δ*t*) of the methods to Equation (17) and investigate under which circumstances the property $\left|Z_{1}^{i}-{\tilde{Z}}_{1}^{i}|<|Z_{0}^{i}-{\tilde{Z}}_{0}^{i}\right|$ is conserved. Here $Z_{1}^{i}$ denotes the approximation to the exact solution $Z^{i}\left(t_{1}\right)$ obtained by the numerical scheme. A straight forward calculation shows that the Euler-Maruyama method retains the property if |1 + λ_*i*_ · Δ*t*/τ| < 1 holds. We conclude that the Euler-Maruyama scheme is stable for $\max_{\lambda_{i}}\zeta_{EM}\left(\lambda_{i}\right)<1$ with ζ_EM_(λ_*i*_) = |1 + λ_*i*_ · Δ*t*/τ|. The scalar exponential Euler method demands a splitting of *A* = −*I* + *W* into −*I* and *W*. Here the stability condition is derived from the modified transformed system

(19) $$\tau dZ^{i}(t)=(-1+{\tilde{\lambda }}_{i})\cdot Z^{i}(t)dt+\sum_{j=1}^{N}\sqrt{\tau }\sigma t^{ij}dW^{j}(t)\;i=1,\ldots ,n$$

where ${\tilde{\lambda }}_{i}$ are the eigenvalues of the matrix *W*. The scalar exponential Euler method conserves the property (Equation 18) if $|e^{-\Delta t/\tau }+{\tilde{\lambda }}_{i}\cdot \left(1-e^{-\Delta t/\tau }\right)|<1$. We conclude that the scalar exponential Euler scheme is stable for $\max_{\lambda_{i}}\zeta_{EXP}\left(\lambda_{i}\right)<1$ with $\zeta_{EXP}\left(\lambda_{i}\right)=|1+\lambda_{i}\cdot \left(1-e^{-\Delta t/\tau }\right)|$.

The implicit Euler method solved with fixed-point iteration is stable, given the convergence of the fixed-point iteration. For the transformed system the employed fixed-point iteration on the single-unit level (Equation 12) reads

$$\Phi (Z_{k+1}^{i,m})=\frac{1}{1+\Delta t/\tau }(Z_{k}^{i}+{\tilde{\lambda }}_{i}Z_{k+1}^{i,m}\Delta t/\tau +\sum_{j=1}^{N}\frac{1}{\sqrt{\tau }}\sigma t^{ij}\Delta W_{k}^{j}).$$

It converges if the scheme Φ is contractive, i.e., if the inequality

$$\left|\Phi (Z_{k+1}^{i,0})-\Phi ({\tilde{Z}}_{k+1}^{i,0})|\overset{!}{<}|(Z_{k+1}^{i,0}-{\tilde{Z}}_{k+1}^{i,0})\right|$$

holds for two different initial values $Z_{k+1}^{i,0}$ and ${\tilde{Z}}_{k+1}^{i,0}$. It follows that the fixed-point iteration converges if $|\frac{\Delta t/\tau }{1+\Delta t/\tau }{\tilde{\lambda }}_{i}|<1$. Thus, the implicit Euler method solved with fixed-point iteration is stable if $\max_{\lambda_{i}}\zeta_{IE}\left(\lambda_{i}\right)<1$ with $\zeta_{IE}\left(\lambda_{i}\right)=\frac{\Delta t/\tau }{1+\Delta t/\tau }|\lambda_{i}+1|$. Hence for all investigated methods the stability depends on the eigenvalues of the matrix *A*, the time constant τ and the step size Δ*t*. To conclude restrictions on the step size Δ*t* we analyze the eigenvalues of *A* for our examples.

For the inhibitory all-to-all test case we determine the eigenvalues $\lambda_{1}=-1-\sqrt{N}$ and λ_2_ = … = λ_*N*_ = −1 of the matrix $A=-I+\frac{-1}{\sqrt{N}}\cdot 𝟙$ analytically. It follows that the Euler-Maruyama scheme satisfies the stability criterion for $\Delta t\leq \frac{2\tau }{\sqrt{N}+1}$, the scalar exponential Euler method demands $\Delta t\leq -\tau \cdot \ln\left(\frac{\sqrt{N}-1}{\sqrt{N}+1}\right)$ and the implicit Euler method with fixed-point iteration requires $\Delta t\leq \frac{\tau }{\sqrt{N}-1}$. For the example of 400 units with τ = 1 in Figure 2A this yields step size restrictions of $\Delta t\leq \frac{2}{21}$ for the Euler-Maruyama method, $\Delta t\leq -\ln\left(\frac{19}{21}\right)\approx 0.1$ for the scalar exponential Euler method and $\Delta t\leq \frac{1}{19}$ for the implicit Euler method. This is consistent with the numerically obtained result (see vertical lines). For all methods the stability criterion implies that the step size Δ*t* needs to be reduced with increasing network size *N* or decreasing time constant τ.

This fully connected network, however, constitutes the worst case test for the class of rate-based models (Equation 9), as the absolute value of the negative eigenvalue quickly increases with the number of units *N*. Our second test case, the sparse balanced e/i network does not suffer from this problem (Rajan and Abbott, 2006), as it is a perfectly balanced network of excitatory and inhibitory units. In a scaling of the connection weights as $\frac{1}{\sqrt{N}}$, the spectral radius of *A* and therefore the subsequent stability analysis is independent of *N*. Here the stability analysis is more complicated, as most of the eigenvalues of *A* are complex and need to be computed numerically.

**Figure 3:**
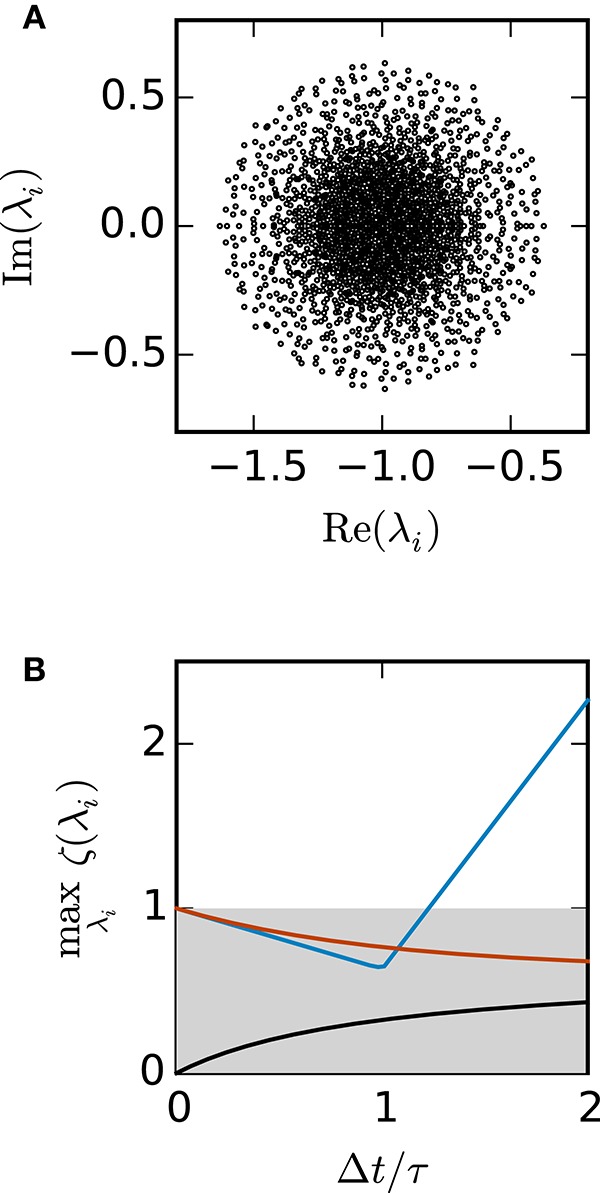
**Stability analysis for the sparse balanced e/i test-case. (A)** Black circles show the eigenvalues λ_*i*_ of the matrix *A*. Network parameters: *N* = 2000, *p* = 0.2. **(B)** The curves show the maximum of the stability function ζ(λ_*i*_) over all eigenvalues λ_*i*_ for the investigated methods (ζ_EM_: blue, ζ_IE_: black, ζ_*EXP*_: red) with respect to Δ*t*/τ. The gray area indicates the region where the stability criterion is met.

Figure 3A shows the eigenvalues of *A* for a network of 2000 units. Figure 3B demonstrates that for this test case the scalar exponential Euler method and the implicit Euler method are stable regardless of the step size Δ*t*. For the Euler-Maruyama the step size is restricted to Δ*t* < 1.2τ. This is again consistent with the results obtained in Figure 2B, where τ = 0.5 and therefore the stability criterion of the Euler-Maruyama method yields Δ*t* < 0.6.

**Figure 4:**
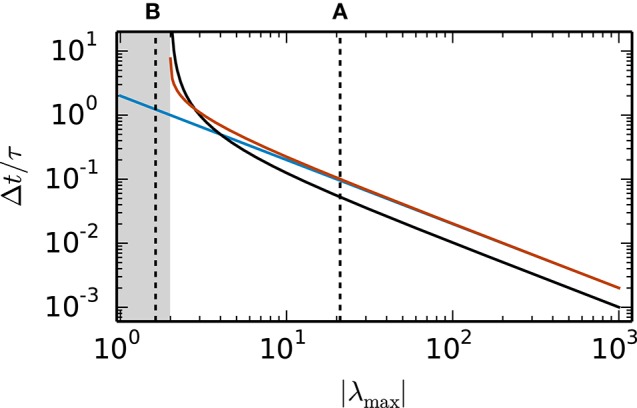
**Step size restrictions of the numerical methods** Largest ratio Δ*t*/τ for which the different numerical methods (blue curve: Euler-Maruyama method, black curve: implicit Euler method solved with fixed-point iteration, red curve: scalar exponential Euler method) are stable shown against the absolute value of λ_max_ = argmax_λ_*i*__ ζ (λ_*i*_) ∈ ℝ. The gray area indicates the region where the scalar exponential Euler method and the implicit Euler method are stable without any restrictions on Δ*t*/τ. The dotted vertical lines correspond to the examples presented in the panels of Figure 2.

Random inhibition-dominated networks exhibit characteristics of both examples. First the matrix *A* contains a real eigenvalue $\lambda_{1}=-1-\alpha \sqrt{N}$ which scales with the network size, however, with a proportionality constant 0 < α < 1 which is reduced compared to the fully connected inhibitory network and determined by the sparseness and the imbalance between excitation and inhibition. Secondly, the matrix *A* contains eigenvalues which constitute a cloud in the complex plane that is determined by the randomness of the connectivity. For these random networks λ_max_ = argmax_λ_*i*__ ζ (λ_*i*_) is a real eigenvalue. Figure 4 shows the step size restrictions of the different numerical methods with respect to the absolute value of λ_max_. For |λ_max_| < 2 the scalar exponential Euler methods and the implicit Euler are stable regardless of the step size Δ*t*. Starting at |λ_max_| ≥ 2.8 the scalar exponential Euler is more stable than the implicit Euler method solved with fixed-point iteration. With increasing |λ_max_| the step size restrictions of the scalar exponential Euler method converges against the step size restriction of the Euler-Maruyama method.

Based on the results in this section we employ the scalar exponential Euler to solve rate-based model dynamics (Equation 9) in our reference implementation. Figure 4 demonstrates that it is the most stable algorithm compatible with the constraints of the distributed simulation scheme for spiking neural networks. Furthermore, the results in Figure 2 indicate that it is the most accurate method in the case of an all-to-all connected network with inhibitory connections. For the sparse balanced excitatory-inhibitory network the analysis exhibits an accuracy similar to the implicit Euler method. However, the solution of the implicit Euler method with fixed-point iteration requires the application of an iterative scheme in each single time step with communication between the units after every iteration. This algorithm is therefore more time consuming than the scalar exponential Euler scheme. Besides the choice of method the analysis in this section indicates that numerical stability is an issue for all tested methods depending on step size Δ*t* and time constant τ. Although the applications in Section 3.3 show that many practical examples do not suffer from stability issues, when a commonly used simulation step size is employed, the inevitable restrictions on the step size Δ*t* should be taken into account in simulations of rate-model networks. For simulations of linear rate models an appropriate step size can be determined by an analysis of the eigenvalues of *A*.

### 3.2. Performance of the reference implementation

This section investigates the performance of the rate model reference implementation. We are interested in i) the scalability of the rate model framework and ii) the comparison between the standard implementation with communication in every computation time step and the iterative approach using waveform relaxation (see Section 2.3.3 for details). We perform the simulations on the JUQUEEN BlueGene/Q supercomputer (Jülich Supercomputing Centre, 2015) at the Jülich Research Centre in Germany. It comprises 28, 672 compute nodes, each with a 16-core IBM PowerPC A2 processor running at 1.6 GHz. For our benchmarks we use 8 OpenMP threads per JUQUEEN compute node and denote by VP = 8 · #nodes the total number of virtual processes employed.

As a test case we employ the sparse balanced e/i test case of linear rate units (ϕ(*x*) = ψ(*x*) = *x*) introduced in Section 3.1, but with a fixed number of 2000 inputs independent of the number of units to allow for an unbiased weak scaling.

**Figure 5:**
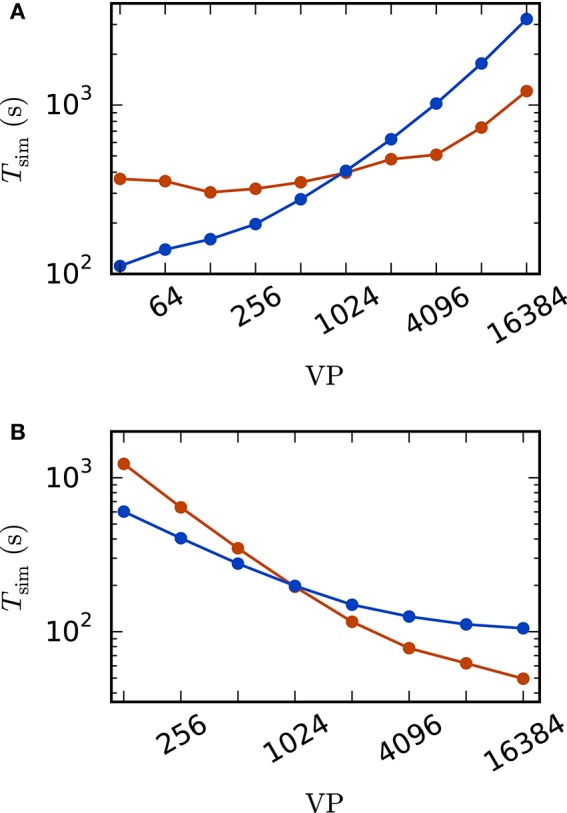
**Scaling behavior of an excitatory-inhibitory network**. Simulation time with waveform relaxation (red curves, wfr_comm_interval: 1.0 ms, wfr_tol: 10^−4^) and without waveform relaxation (blue curves) as a function of the number of virtual processes in double logarithmic representation. The simulations span *T* = 100 ms of biological time at a computation step size of *h* = 0.1 ms. Sparse balanced e/i test case but with a fixed number of 2,000 inputs per unit. Other parameters: μ = 0, σ = 1 and τ = 10 ms **(A)** Weak scaling with 100 units per virtual process VP. **(B)** Strong scaling with a total number of *N* = 51, 200 units.

A weak scaling (Figure 5A) shows that the scalability of the standard implementation is impaired by the massive amount of communication. While for perfect scaling the simulation time should be constant over the number of virtual processes, the actual simulation time is increased by 15–25% when the number of virtual processes is doubled for VP < 256 and even up to 83% from 8, 192 to 16, 384 virtual processes. For the iterative method, the scaling behavior is close to constant up to 1, 024 virtual processes. When more processes are employed, the simulation time is increasing. However, the iterative method shows a better scaling behavior as the increase is weaker compared to the standard computation due to the lower total number of communication steps. Due to the higher computational load of the iterative method (see Section 2.3.3) the simulation time is larger compared to the straight forward approach for a small number of VP, where communication is not that crucial. For VP ≥ 1024, the iterative approach is superior with a speed up factor close to three for 16, 384 virtual processes (1, 209 s vs. 3, 231 s).

The strong scaling scenario with a fixed total number of *N* = 51, 200 units in Figure 5B constitutes a similar result. The iterative approach is beneficial for more than 1, 024 virtual processes and the scaling behavior of the iterative method outperforms that of the standard computation. Starting at 4, 096 virtual processes the savings in computation time decrease, which is explained by the very low workload of each single compute node. Again, for a smaller number of virtual processes the amount of additional computations is too high to outperform the standard implementation.

Despite the overall good scaling behavior, the performance in terms of absolute compute time is inferior to a simulator specifically designed for rate-based models alone (not shown). In the latter case it increases performance to collect the states of all units in one vector. If further the connectivity is available in form of a matrix and the delays are zero or homogeneous, the network can be efficiently updated with a single matrix-vector multiplication. Thus, the increased functionality and flexibility of having rate- and spiking models unified in one simulator comes for the price of a loss of performance for the rate-based models. However, as noted in the introduction, the number of units in rate-based network models is usually small and therefore performance is not as critical as for spiking network models.

### 3.3. Applications

First, we discuss a random inhibition-dominated network of linear rate units, then include nonlinear rate dynamics in a random network, and spatially structured connectivity in a functional neural-field model. In each case, simulation results are compared to analytical predictions. Furthermore, we simulate a mean-field model of a spiking model of a cortical microcircuit and discuss possible generalizations.

#### 3.3.1. Linear model

In the asynchronous irregular regime which resembles cortical activity, the dominant contribution to correlations in networks of nonlinear units is given by effective interactions between linear response modes (Pernice et al., 2011; Trousdale et al., 2012; Grytskyy et al., 2013; Dahmen et al., 2016). Networks of such noisy linear rate models have been investigated to explain features such as oscillations (Bos et al., 2016) or the smallness of average correlations (Tetzlaff et al., 2012; Helias et al., 2013). We here consider a prototypical network model of excitatory and inhibitory units following the linear dynamics given by Equation 9 with ϕ(*x*) = ψ(*x*) = *x*, μ = 0, and noise amplitude σ,

(20) $$\tau dX^{i}(t)=\left(-X^{i}+\sum_{j=1}^{N}w^{ij}X^{j}(t)\right)dt+\sqrt{\tau }\sigma dW^{i}(t).$$

**Figure 6:**
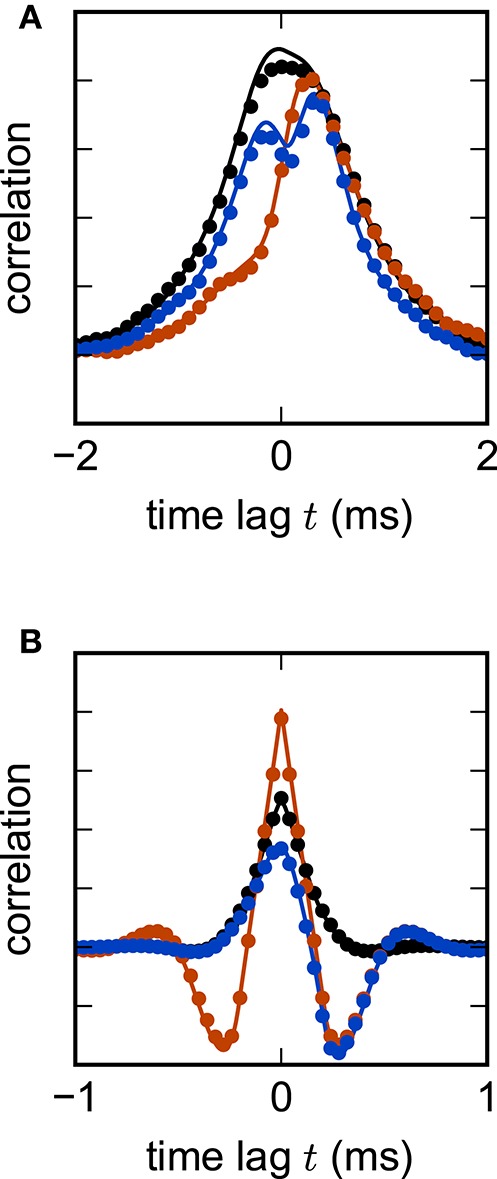
**Linear rate model of a random excitatory-inhibitory network. (A)** Cross-correlation functions of two pairs of excitatory units (black, red) and an excitatory-inhibitory unit pair (blue) in a network without delay. The variability across correlation functions arises from heterogeneity in network connections (difference between black and red curves) and from different combinations of cell types (e.g., difference between black and blue curves). **(B)** Population-averaged autocorrelation function for excitatory (black) and inhibitory units (red), and cross-correlation function between excitatory and inhibitory units (blue) in a network with delay *d* = 2 ms. Symbols denote simulation results, curves show theoretical predictions. Parameters: *N*_*E*_ = 80 excitatory and *N*_*I*_ = 20 inhibitory units, random connections with fixed out-degree, connection probability *p* = 0.1, excitatory weight $w_{E}=1/\sqrt{N_{E}+N_{I}}$, inhibitory weight *w*_*I*_ = −6*w*_*E*_, τ = 1 ms, μ = 0, σ = 1. Step size *h* = 0.1 ms.

Due to the linearity of the model, the cross-covariance between units *i* and *j* can be calculated analytically and is given by Ginzburg and Sompolinsky (1994); Risken (1996); Gardiner (2004); Dahmen et al. (2016):

(21) $$c(t)=\sum_{i,j}\frac{v^{i\text{T}}\sigma^{2}v^{j}}{\lambda_{i}+\lambda_{j}}u^{i}u^{j\text{T}}\left(H(t)\frac{1}{\tau }e^{-\lambda_{i}\frac{t}{\tau }}+H(-t)\frac{1}{\tau }e^{\lambda_{j}\frac{t}{\tau }}\right),$$

where *H* denotes the Heaviside function. The λ_*i*_ indicate the eigenvalues of the matrix 𝟙 − *W* corresponding to the *i*-th left and right eigenvectors *v*^*i*^ and *u*^*i*^ respectively. Nonzero delays yield more complex analytical expressions for cross-correlations. In the population-averaged case, theoretical predictions are still analytically tractable (Equation 18 in Grytskyy et al., 2013). Figure 6 shows the cross-covariance functions for pairs of instantaneously coupled units in a large network, as well as population-averaged covariance functions in a network of excitatory and inhibitory units with delayed interactions. In both cases, simulations are in good agreement with the theoretical predictions.

#### 3.3.2. Non-linear model

**Figure 7:**
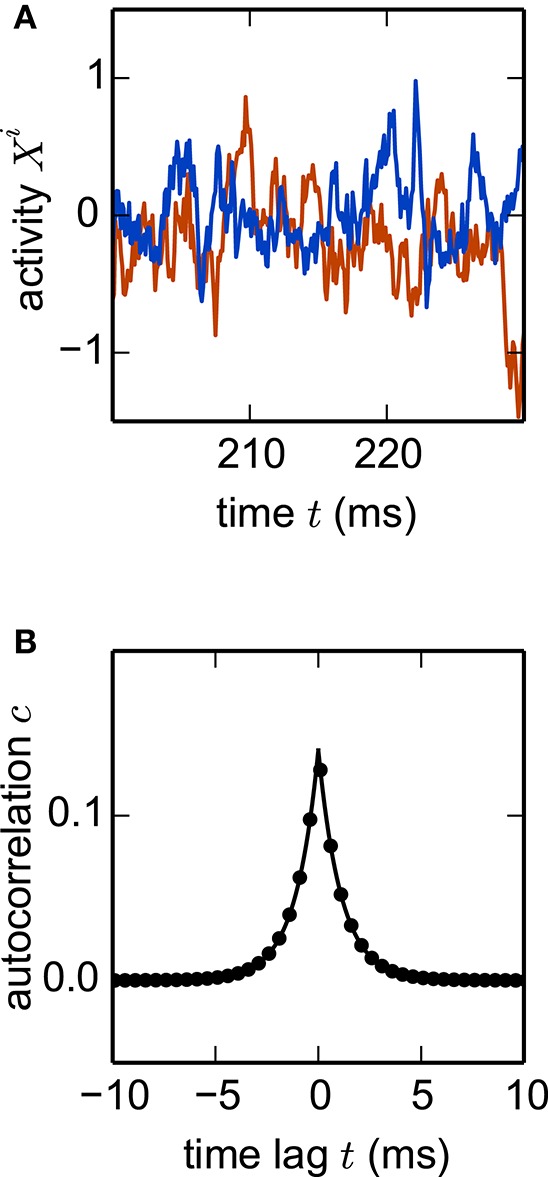
**Nonlinear network model**. Simulation of the network specified by Equation (22) with *N* = 1000 units. **(A)** Noisy example trajectories of two units. **(B)** Autocorrelation function obtained by simulation averaged over all units (dots) and theory (solid curve). Other parameters: τ = 1 ms, σ = 0.5, *g* = 0.5. Step size *h* = 0.1 ms.

So far we considered a network with linear couplings between the units. Qualitatively new features appear in the presence of nonlinearities. One of the most prominent examples is the emergence of chaotic dynamics (Sompolinsky et al., 1988) in a network of nonlinearly coupled rate units. The original model is deterministic and has been recently extended to stochastic dynamics (Goedeke et al., 2016). The model definition follows from Equation (9) with μ = 0, ϕ(*x*) = *x*, ψ(*x*) = tanh(*x*), i.e.

(22) $$\tau dX^{i}(t)=\left(-X^{i}(t)+\sum_{j=1}^{N}w^{ij}\tanh\left(X^{j}(t)\right)\right)dt+\sqrt{\tau }\sigma dW^{i}(t),$$

where $w^{ij}\approx N\left(0,g^{2}/N\right)$ are Gaussian random couplings. In the thermodynamic limit *N* → ∞, the population averaged autocorrelation function *c*(*t*) can be determined within dynamic mean-field theory (Sompolinsky et al., 1988; Goedeke et al., 2016; Schuecker et al., 2016). Comparing *c*(*t*) obtained by simulation of a network (Equation 22) with the analytical result (Goedeke et al., 2016, their Equations 6 and 8) demonstrates excellent agreement (Figure 7). The simulated network is two orders of magnitude smaller than the cortical microcircuit, illustrating that in this context finite-size effects are already negligible at this scale.

#### 3.3.3. Functional model

Complex dynamics not only arises from nonlinear single-unit dynamics, but also from structured network connectivity (Yger et al., 2011). One important nonrandom feature of brain connectivity is the spatial organization of connections (Malach et al., 1993; Voges et al., 2010). In spatially structured networks, delays play an essential role in shaping the collective dynamics (Roxin et al., 2005; Voges and Perrinet, 2012). Patterns of activity in such networks are routinely investigated using neural-field models. In contrast to the models discussed above, field models require a discretization of space for numerical simulation. Such discretization can be done in the real space, leading effectively to a network of units at discrete positions in space, or alternatively, for particular symmetries in the couplings, in k-space (Roxin et al., 2006). Here, we follow the more general approach of discretization in real space.

A prototypical model of a spatial network is given by Roxin et al. (2005), where the authors consider the neural-field model

(23) $$\begin{array}{l} \tau dX(\varphi ,t)=\\ \left(-X(\varphi ,t)+\phi \left[I_{\text{ext}}+\int_{-\pi }^{\pi }d\varphi \prime w(|\varphi -\varphi \prime |)X(\varphi \prime ,t-d)\right]\right)dt \end{array}$$

with delayed (delay *d*) interactions, constant input *I*_ext_, threshold-linear activation function ϕ = *x* · *H*(*x*) and periodic Mexican-hat shaped connectivity

(24) $$w(|\varphi -\varphi^{\prime }|)=w_{0}+w_{1}\cos(\varphi -\varphi^{\prime }).$$

The spatial variable φ can also be interpreted as the preferred orientation of a set of units, thus rendering Equation (23) a model in feature space (Hansel and Sompolinsky, 1998). Discretizing space into *N* segments yields the following set of coupled ODEs:

(25) $$\tau dX^{i}=\left(-X^{i}+\phi \left[I_{\text{ext}}+\sum_{j=1}^{N}w^{ij}X^{j}(t-d)\right]\right)dt$$

with connectivity $w^{ij}=\frac{2\pi }{N}w\left(\left|\varphi^{i}-\varphi^{j}\right|\right)$, $\varphi_{i}=-\pi +\frac{2\pi }{N}\cdot i$ for *i* ∈ [1, *N*] and discretization factor $\frac{2\pi }{N}$ that scales the space constants *w*_0_ and *w*_1_ with the neuron density. The spatial connectivity together with a delay in the interaction introduce various spatial activity patterns depending on the shape of the Mexican-hat connectivity.

**Figure 8:**
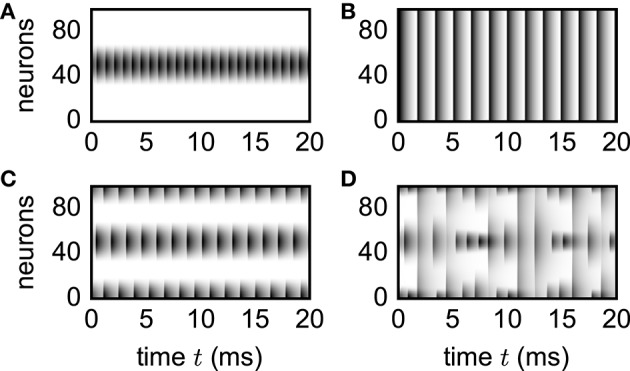
**Spatial patterns in functional neural-field model**. Vertical axis shows unit indices organized according to ascending angle φ ∈ [−π, π). Activity *X*_*i*_(*t*) = *X*(φ_*i*_, *t*) encoded by gray scale with white denoting no activity. Initial transients not shown. Patterns reproduce the analytically derived phase diagram in the original study by Roxin et al. (2005). Parameters: *N* = 100, *d* = 0.1 ms, τ = 1 ms, *I*_ext_ = 1, *w*_0_ = −80, *w*_1_ = 15 **(A)**, *w*_1_ = 5 **(B)**, *w*_1_ = −46 **(C)**, *w*_1_ = −86 **(D)**. Initial condition: $X_{i}\left(0\right)=X\left(\varphi_{i},0\right)=\pi^{2}-\varphi_{i}^{2}$. Step size *h* = 0.01 ms.

To illustrate applicability of the simulation framework to neural-field models, we reproduce various patterns (Figure 8) observed by Roxin et al. (2005). Although the discrete and continuous networks strictly coincide only in the thermodynamic limit *N* → ∞, numerically obtained patterns shown in Figure 8 well agree with the analytically derived phase diagram of the continuous model (Roxin et al., 2005) already for network sizes of only *N* = 100 units.

#### 3.3.4. Mean-field analysis of complex networks

A network of spiking neurons constitutes a high dimensional and complex system. To investigate its stationary state, one can describe the activity in terms of averages across neurons and time, leading to population averaged stationary firing rates (Brunel, 2000). Here, the spatial average collapses a large number of neurons into a single population, which is interpreted as a single rate unit. The ability to represent spiking as well as rate dynamics by the same simulation framework allows a straight-forward analysis of the spiking network by replacing the spiking neuron populations by single rate-based units.

In more formal terms, we now consider networks of neurons structured into *N* interconnected populations. A neuron in population α receives *K*_αβ_ incoming connections from neurons in population β, each with synaptic efficacy *w*_αβ_. Additionally, each neuron in population α is driven by *K*_α,ext_ Poisson sources with rate *X*_ext_ and synaptic efficacy *w*_ext_. We assume leaky integrate-and-fire model neurons with exponentially decaying post-synaptic currents. The dynamics of membrane potential *V* and synaptic current *I*_s_ is (Fourcaud and Brunel, 2002)

(26) $$\begin{array}{l} \tau_{\text{m}}\frac{dV^{i}}{dt}=-V^{i}+I_{\text{s}}^{i}\\ \;\tau_{\text{s}}\frac{dI_{\text{s}}^{i}}{dt}=-I_{\text{s}}^{i}+\tau_{\text{m}}\sum_{j=1}^{N}w^{ij}\sum_{k}\delta (t-t_{k}^{j}-d), \end{array}$$

where $t_{k}^{j}$ denotes the *k*-th spike-time of neuron *j*, and τ_m_ and τ_s_ are the time constants of membrane and synapse, respectively. The membrane resistance has been absorbed in the definition of the current. Whenever the membrane potential *V* crosses the threshold θ, the neuron emits a spike and *V* is reset to the potential *V*_r_, where it is clamped for a period of length τ_r_. Given that all neurons have identical parameters, a diffusion approximation, assuming asynchronous and Poissonian spiking statistics as well as small synaptic couplings, leads to the population-averaged firing rates *X*_α_ (Fourcaud and Brunel, 2002)

(27) $$\begin{array}{l} \frac{1}{X_{\alpha }}=\tau_{\text{r}}+\tau_{\text{m}}\sqrt{\pi }\int_{(V_{\text{r}}-\mu_{\alpha })/\sigma_{\alpha }+\gamma \sqrt{\tau_{\text{s}}/\tau_{\text{m}}}}^{(\theta -\mu_{\alpha })/\sigma_{\alpha }+\gamma \sqrt{\tau_{\text{s}}/\tau_{\text{m}}}}e^{u^{2}}\;(1+\text{erf}(u))\text{d}u\\ \;=\text{​​}:1/\Phi_{\alpha }(X) \end{array}$$

(28) $$\mu_{\alpha }=\;\tau_{\text{m}}\sum_{\beta }K_{\alpha \beta }w_{\alpha \beta }X_{\beta }+\tau_{\text{m}}K_{\alpha ,\text{ext}}w_{\text{ext}}X_{\text{ext}}$$

(29) $$\sigma_{\alpha }^{2}=\tau_{\text{m}}\sum_{\beta }K_{\alpha \beta }w_{\alpha \beta }^{2}X_{\beta }+\tau_{\text{m}}K_{\alpha ,\text{ext}}w_{\text{ext}}^{2}X_{\text{ext}}.$$

Here, $\gamma =|\zeta \left(1/2\right)|/\sqrt{2},$ with ζ denoting the Riemann zeta function (Abramowitz and Stegun, 1974). We find the fixed points of Equation (27) by solving the first-order differential equation (Wong and Wang, 2006; Schuecker et al., 2017)

(30) $$\tau \frac{\text{d}X_{\alpha }}{dt}=-X_{\alpha }+\Phi_{\alpha }(X),$$

which constitutes a network of rate units with the dimension equal to the number of populations *N*.

**Figure 9:**
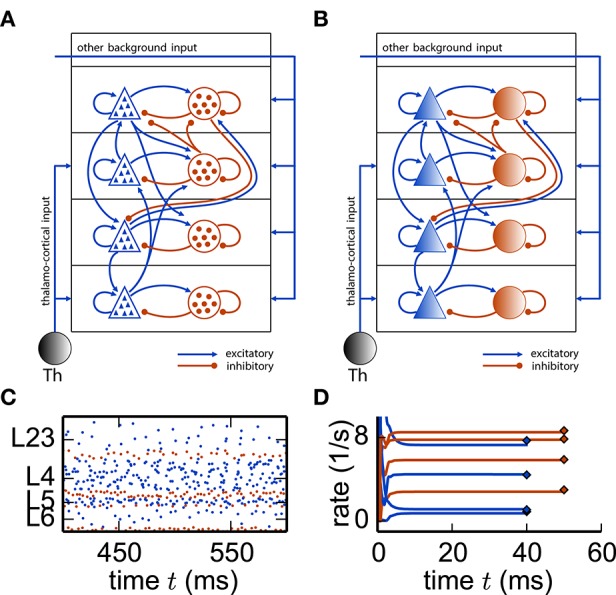
**Reduction of spiking microcircuit model to rate dynamics. (A)** Sketch of a microcircuit model (Potjans and Diesmann, 2014) with excitatory (blue triangles) and inhibitory (red disks) neuron populations, each consisting of a large number of neurons indicated by the small triangles and disks respectively. Arrows between populations indicate the in-degree *K*. **(B)** Sketch of the corresponding reduced model where each population is replaced by a single rate unit. **(C)** Spiking activity in the different layers. **(D)** Dynamics of the eight units of the rate network (Equation 30) (curves) and comparison to population-averaged firing rates obtained from direct simulations of the spiking network (diamonds).

Next we apply this framework to a cortical microcircuit model (Potjans and Diesmann, 2014) constituting roughly 80, 000 spiking neurons structured into 8 populations across 4 layers [*L*23, *L*4, *L*5, *L*6], with one excitatory and one inhibitory cell type each (Figure 9). The model exhibits irregular and stationary spiking activity (Figure 9C). Replacing each population by a single rate unit (Figure 9B) results in an eight-dimensional rate network (Equation 30) which converges to a fixed point corresponding to the population-averaged firing rates obtained from direct simulation of the spiking model (Figure 9D).

The analysis only considers the stationary state of the microcircuit, which can as well be determined using the population-density approach (Cain et al., 2016). While the mean-field approach presented is strictly valid only in the thermodynamic limit, finite-size fluctuations around this state are accessible using the noisy linear-rate model (Section 3.3.1) as elaborated by Bos et al. (2016) or within the population-density approach (Schwalger et al., 2016).

#### 3.3.5. Further rate models

##### 3.3.5.1. Non-linear dynamics

A characteristic feature of rate models considered so far is the leaky dynamics, i.e., the linear term −*X*^*i*^(*t*) in Equation (9). However, the presented framework can be extended to nonlinear dynamics as used for example by Stern et al. (2014). In a more general form Equation (9) reads

(31) $$\begin{array}{l} \tau dX^{i}(t)=\left[a(X^{i}(t))+\phi \left(\sum_{j=1}^{N}w^{ij}\psi \left(X^{j}(t-d)\right)\right)\right]dt\\ \;+\;\sqrt{\tau }\sigma dW^{i}(t) \end{array}$$

where *a* characterizes the intrinsic rate dynamics. If *a* does not contain a linear part, the Euler-Maruyama scheme can be used for the update, i.e.,

(32) $$\begin{array}{l} X_{k+1}^{i}=X_{k}^{i}+\left[a(X_{k}^{i})+\phi \left(\sum_{j=1}^{N}w^{ij}\psi \left(X_{k-\frac{d}{\Delta t}}^{j}\right)\right)\right]\frac{1}{\tau }\Delta t\\ \;+\;\frac{1}{\sqrt{\tau }}\sigma \Delta W_{k}^{i}. \end{array}$$

If *a* also contains a linear part, so that *a*(*X*^*i*^) = −*X*^*i*^ + *f*(*X*^*i*^), one can use an exponential Euler update approximating the nonlinear part as constant during the update. This leads to

(33) $$\begin{array}{l} X_{k+1}^{i}=e^{-\Delta t/\tau }X_{k}^{i}\;+\;\left(1-e^{-\Delta t/\tau }\right)\\ \;\left[f(X_{k}^{i})\;+\;\phi \left(\sum_{j=1}^{N}w^{ij}\psi \left(X_{k-\frac{d}{\Delta t}}^{j}\right)\right)\right]\\ \;+\;\sqrt{\frac{1}{2}(1-e^{-2\Delta t/\tau })}\sigma \eta_{k}^{i}, \end{array}$$

with $\eta_{k}^{i}\sim N\left(0,1\right).$

##### 3.3.5.2. Multiplicative coupling

Another possible extension is a multiplicative coupling between units as for example employed in Gancarz and Grossberg (1998) or the original works of Wilson and Cowan (1972, 1973). In the most general form, this amounts to

(34) $$\begin{array}{l} \tau dX^{i}(t)=\left[-X^{i}(t)+H(X^{i})\cdot \phi \left(\sum_{j=1}^{N}w^{ij}\psi \left(X^{j}(t-d)\right)\right)\right]dt\\ \;+\;\sqrt{\tau }\sigma dW^{i}(t), \end{array}$$

which, again assuming the coupling term to be constant during the update, can be solved using the exponential Euler update

(35) $$\begin{array}{l} X_{k+1}^{i}=e^{-\Delta t/\tau }X_{k}^{i}\;+\;\left(1-e^{-\Delta t/\tau }\right)\\ \;\left[H(X_{k}^{i})\cdot \phi \left(\sum_{j=1}^{N}w^{ij}\psi \left(X_{k-\frac{d}{\Delta t}}^{j}\right)\right)\right]\\ \;+\;\sqrt{\frac{1}{2}(1-e^{-2\Delta t/\tau })}\sigma \eta_{k}^{i}, \end{array}$$

with $\eta_{k}^{i}\sim N\left(0,1\right).$

##### 3.3.5.3. Multiplicative noise

So far, we have considered rate models subject to additive noise corresponding to *b*(*t, x*(*t*)) = *b*(*t*) in Equation (4). The linear rate model considered in Section 3.3.1 describes the dynamics around a stationary state and due to the stationary baseline, the noise amplitude is constant. However, one might relax the stationarity assumption which would render the noise amplitude proportional to the time dependent rate, i.e., a multiplicative noise amplitude. The presented framework covers the latter since the exponential Euler update is also valid for multiplicative noise (Equation 8).

##### 3.3.5.4. Output noise

Grytskyy et al. (2013) show that there is a mapping between a network of leaky integrate-and-fire models and a network of linear rate models with so-called output noise. Here the noise is added to the output rate of the afferent units

(36) $$\begin{array}{l} \tau \frac{dX^{i}(t)}{dt}=-X^{i}(t)+\mu +\phi \text{​​}\left(\sum_{j=1}^{N}w^{ij}\psi \left(X^{j}(t-d)+\sqrt{\tau }\sigma \xi^{j}(t)\right)\text{​}\right)\\ \;i=1,\ldots ,N \end{array}$$

and we cannot write the system as a SDE of type (2), as the nonlinearities ϕ(*x*) and ψ(*x*) are also applied to the white noise ξ^*j*^. In addition to the implementation rate_neuron_ipn for the rate-based models (Equation 9) discussed in the present work, our reference implementation also contains a base implementation rate_neuron_opn for models with output noise. For these models, the stochastic exponential Euler method can not be employed. Instead the solver assumes the noise ξ^*j*^ to be constant over the update interval which leads to the update formula

(37) $$\begin{array}{l} X_{k+1}^{i}=e^{-\Delta t/\tau }X_{k}^{i}\;+\;\left(1-e^{-\Delta t/\tau }\right)\\ \;\left[\mu +\phi \left(\sum_{j=1}^{N}w^{ij}\psi \left(X_{k}^{j}\;+\;\sqrt{\frac{\tau }{\Delta t}}\sigma \eta_{k}^{j}\right)\right)\right]. \end{array}$$

The term $X_{k}^{j}+\sqrt{\frac{\tau }{\Delta t}}\sigma \eta_{k}^{j}$ with $\eta_{k}^{j}\sim N\left(0,1\right)$ is calculated beforehand in the sending unit *j*, which results in the same amount of communicated data as in the case of models with input noise.

## 4. Discussion

This work presents an efficient way to integrate rate-based models in a neuronal network simulator that is originally designed for models with delayed spike-based interactions. The advantage of the latter is a decoupling of neuron dynamics between spike events. This is used by current parallel simulators for large-scale networks of spiking neurons to reduce communication between simulation processes and significantly increase performance and scaling capabilities up to supercomputers (Morrison et al., 2005). In contrast, rate-based models interact in a continuous way. For delayed interactions, rate dynamics are still decoupled for the minimal delay of the network such that information can be exchanged on a coarse time-grid. For instantaneous coupling, communication in every time step is required. This is feasible for small networks that can be simulated on small machines and thus require only a small amount of communication. For improved efficiency of simulations of large networks on supercomputers, we implement an iterative numerical solution scheme (Lelarasmee, 1982). Furthermore, we investigate several standard methods for the solution of rate model equations and demonstrate that the scalar exponential Euler method is the best choice in the context of a neuronal network simulator that is originally designed for models with delayed spike-based interactions. Afterwards, we show the applicability of the numerical implementation to a variety of well-known and widely-used rate models and illustrate possible generalizations to other categories of rate-based models.

The current reference implementation uses an exponential Euler scheme (Adamu, 2011; Komori and Burrage, 2014) with a diagonal matrix *A* (scalar exponential Euler): The additive noise as well as the leaky dynamics of single neurons are exactly integrated while the network input to the rate units is approximated as piecewise constant. The analysis in Section 3.1 demonstrates that the scalar exponential Euler is the most accurate, stable and efficient standard-method for SDEs that is applicable to a distributed spiking simulator. In particular for all-to-all connected networks of linear rate units the distributed design renders implicit methods less feasible, as the convergence of the involved fixed-point iteration requires small time-steps. For all methods the computation step size needs to be compared against the time constant τ. Therefore, stable solutions for small values τ ≪ 1 may require to decrease the step size below a default value.

The reference implementation provides an optional iterative method, the waveform relaxation (Lelarasmee, 1982), for networks with instantaneous rate connections. This method obtains the same results as the standard approach, but improves scalability by reducing communication at the cost of additional computations. As a consequence, the optimal method (standard vs. iterative) depends on the numbers of compute nodes and virtual processes. In our test case the use of the waveform-relaxation technique is beneficial for 1024 or more virtual processes. It is therefore recommended to employ the iterative scheme for large-scale simulations on supercomputers, but to disable it for smaller rate-model simulations on local workstations or laptops. This can easily be achieved by the parameter use_wfr (see Section 2.3.3 for details) of the algorithm. In general, the scalability for simulations of rate models is worse than for spiking network simulations (Kunkel et al., 2014) and comparable to simulations with gap junctions (Hahne et al., 2015). This is expected since for rate connections as well as for gap junctions a large amount of data needs to be communicated compared to a spiking simulation. Future work should assess whether this bottleneck can be overcome by a further optimized communication scheme.

While our reference implementation uses the simulation software NEST as a platform, the employed algorithms can be ported to other parallel spiking network simulators. Furthermore, the implementation of the example rate models as templates allows customization to arbitrary gain functions. Researchers can create additional models, without in-depth knowledge of simulator specific data structures or numerical methods. In addition, the infrastructure is sufficiently general to allow for extensions to other categories of rate models as shown explicitly for nonlinear dynamics, multiplicative coupling, and other types of noise. This design enables the usage of the framework for a large body of rate-based network models. Furthermore, the generality of the model equations supports applications beyond neuronal networks, such as in computational gliascience (Amiri et al., 2012) or artificial intelligence (Haykin, 2009).

Some design decisions for the reference implementation come with an up- and a downside and may at the present state of knowledge and experience constitute judgment calls: The choice to determine the type of nonlinearity of the recurrent network input with a boolean parameter is based on the assumption that this implementation covers the majority of rate models used in neuroscience today. The solution has an advantage in maintainability as it results in half as many template instances for a given set of gain functions than the alternative solution discussed above. It also avoids the introduction of potentially confusing names of rate models encoding the nature of the nonlinearity. On the downside models that do actually employ both nonlinearities at once cannot be expressed. Furthermore, a decision that can already be made at the time when the model instance is created, is delayed to the simulation phase. The decision to create a separate connection type for mean-field models of the siegert type is led by the ambition to avoid memory overhead. This comes at the price that units of this type cannot be connected to instances of rate models using the generic rate connection. Adapter elements like the parrot_neuron (see Kunkel et al., 2011, for a recent application) are one way to overcome this problem. Only the experience of researchers with the present implementation will inform us on whether characteristics and user interface serve the purpose of the community or if particular decisions need revision.

Mean-field theory has built a bridge between networks of spiking neurons and rate-based units that either represent single neurons or populations (Buice and Chow, 2007; Buice et al., 2010; Ostojic and Brunel, 2011; Bressloff, 2012; Grytskyy et al., 2013). In the latter case, the rate-based approach comes along with a considerable reduction of dimensionality (Section 3.3.4). Due to a possibly large number of populations, the fixed-point solution of the stationary activity can generally not be determined analytically, but still be found by evolving a pseudo-time dynamics. Within the presented framework, this approach is much faster than the spiking counter-part and thus facilitates the calibration of large-scale spiking network models (Schuecker et al., 2017).

Our unifying framework allows researchers to easily switch between rate-based and spiking models in a particular network model requiring only minimal changes to the simulation script. This facilitates an evaluation of the different model types against each other and increases reproducibility in the validation of reductions of spiking networks to rate-based models. Furthermore, it is instructive to study whether and how the network dynamics changes with the neuron model (Brette, 2015). In particular, functional networks being able to perform a given task are typically designed with rate-based units. Their validity can now be evaluated by going from a more abstract rate-based model to a biologically more realistic spiking neuron model. The present reference implementation does not allow for interactions between spiking and rate-based units. While this is technically trivial to implement, the proper conversion from spikes to rates and vice versa is a conceptual issue that has to be explored further by theoretical neuroscience.

The presented joined platform for spike-based and rate-based models hopefully triggers new research questions by facilitating collaboration and translation of ideas between scientists working in the two fields. This work therefore contributes to the unification of both modeling routes in multi-scale approaches combining large-scale spiking networks with functionally inspired rate-based elements to decipher the dynamics of the brain.

## Author contributions

Under the supervision of MH and MD, the authors JH, DD, and JS jointly worked on all parts of the above publication. DD and JS thereby focused on the neuroscientific applications and the implementation of neuron models. JH focused on the NEST infrastructure, numerical schemes and performance benchmarks. All authors contributed to the writing of the manuscript.

### Conflict of interest statement

The authors declare that the research was conducted in the absence of any commercial or financial relationships that could be construed as a potential conflict of interest.

## A. Appendix

### A.1. numerical evaluation of the siegert formula

We here describe how to numerically calculate Equation (27), frequently called Siegert formula in the literature (for a recent textbook see Gerstner et al., 2014), which is not straight forward due to numerical instabilities in the integral. First we introduce the abbreviations $y_{\theta }=\left(\theta -\mu \right)/\sigma +\gamma \sqrt{\tau_{s}/\tau_{m}}$ and $y_{r}=\left(V_{r}-\mu \right)/\sigma +\gamma \sqrt{\tau_{s}/\tau_{m}}$ and rewrite the integral as

$$\begin{array}{l} \sqrt{\pi }\int_{y_{r}}^{y_{\theta }}e^{u^{2}}(1+\text{erf}(u))du\\ =2\int_{y_{r}}^{y_{\theta }}\;e^{u^{2}}\int_{-\infty }^{u}e^{-v^{2}}\;dv\;du\\ =2\int_{y_{r}}^{y_{\theta }}\;\int_{-\infty }^{u}e^{\left(u+v)(u-v\right)}\;dv\;du. \end{array}$$

Here, the numerical difficulty arises due to a multiplication of a divergent (*e*^*u*^2^^) and a convergent term (1 + erf(*u*)) in the integrand. We therefore use the variable transform *w* = *v* − *u* and obtain

$$\begin{array}{l} =\;2\int_{y_{r}}^{y_{\theta }}\;\int_{-\infty }^{u}e^{\left(u+v)(u-v\right)}\;dv\;du\\ =2\int_{y_{r}}^{y_{\theta }}\;\int_{-\infty }^{0}e^{\left(2u+w)(-w\right)}\;dw\;du\\ =\;\int_{0}^{\infty }e^{-w^{2}}\frac{e^{2y_{\theta }w}-e^{2y_{r}w}}{w}\;dw, \end{array}$$

where we performed the integral over *u* in the last line.

For *y*_*r*_, *y*_θ_ < 0 the integrand can be integrated straightforwardly as

(A1) $$\int_{0}^{\infty }\frac{e^{2y_{\theta }w-w^{2}}-e^{2y_{r}w-w^{2}}}{w}\;dw\;,$$

where the two terms in the integrand converge separately and where, in approximation, the upper integration bound is chosen, such that the integrand has dropped to a sufficiently small value (here chosen to be 10^−12^). Here, for *w* = 0, the integrand has to be replaced by $\lim_{w\to 0}\frac{e^{2y_{\theta }w}-e^{2y_{r}w}}{w}=2\left(y_{\theta }-y_{r}\right)$.

For *y*_θ_ > 0 and *y*_*r*_ < 0 only the combination of the two terms in Equation (A1) converges. So we rewrite

(A2) $$\begin{array}{l} \;\int_{0}^{\infty }e^{-w^{2}}\frac{e^{2y_{\theta }w}-e^{2y_{r}w}}{u}\;dw\\ =\int_{0}^{\infty }e^{2y_{\theta }w-w^{2}}\frac{1-e^{2(y_{r}-y_{\theta })w}}{w}\;dw\\ =\;e^{y_{\theta }^{2}}\int_{0}^{\infty }e^{-{\left(w-y_{\theta }\right)}^{2}}\frac{1-e^{2(y_{r}-y_{\theta })w}}{w}\;dw. \end{array}$$

The integrand has a peak near *y*_θ_. Therefore, in approximation, the lower and the upper boundary can be chosen to be left and right of *y*_θ_, respectively, such that the integrand has fallen to a sufficiently low value (here chosen to be 10^−12^). For *w* = 0 we replace the integrand by its limit, which is $\lim_{w\to 0}e^{-{\left(w-y_{\theta }\right)}^{2}}\frac{1-e^{2\left(y_{r}-y_{\theta }\right)w}}{w}=e^{-y_{\theta }^{2}}2\left(y_{\theta }-y_{r}\right)$.

We actually switch from Equations (A1) to (A2) when *y*_θ_ > 0.05|Ṽ_θ_|/σ_α_ with ${Ṽ}_{\theta }=V_{\theta }+\gamma \sqrt{\tau_{s}/\tau_{m}}$. This provides a numerically stable solution in terms of a continuous transition between the two expressions. Our reference implementation numerically evaluates the integrals using the adaptive GSL implementation gsl_integration_qags (Galassi et al., 2006) of the Gauss-Kronrod 21-point integration rule.

### A.2. usage of the NEST reference implementation

We here give a brief description of how to use our reference implementation of the continuous-time dynamics in the simulation code NEST with the syntax of the PyNEST interface (Eppler et al., 2009). Complete simulation scripts will be made available with one of the next major releases of NEST. The description focuses on rate-model specific aspects. A general introduction to PyNEST can be found on the simulator website (http://www.nest-simulator.org).

Script 1 shows the creation of an excitatory-inhibitory network of linear rate units as used in our example in Section 3.3.1. Researchers already familiar with the PyNEST interface notice that there is no fundamental difference to scripts for the simulation of spiking neural networks. Line 4 illustrates how to disable the usage of the waveform-relaxation method. This is advisable for simulations on local workstations or clusters, where the waveform-relaxation method typically does not improve performance (see Section 3.2). The iterative method is enabled by default, as it is also employed for simulations with gap junctions, where it improves performance and even accuracy of the simulation, regardless of network size and parallelization (Hahne et al., 2015). Instances of the linear rate-based model are created by calling nest.Create in the usual way with model type lin_rate_ipn. The parameter linear_summation characterizes the type of nonlinearity (ϕ or ψ, see Section 2.3.2) of the rate model. In this particular example the explicit specification of the parameter is added for illustrative purposes only as i) the employed model is a linear rate model, where regardless of the choice of the parameter ϕ(*x*) = ψ(*x*) = *x* holds and ii) the default value is True anyway. Lines 13-14 and 31 demonstrate how to record the rate activity with the multimeter. The record_from parameter needs to be set to rate to pick up the corresponding state variable. As this particular network model includes delayed rate connections the synapse model delay_rate_connection is chosen (lines 17–20). In order to create instantaneous rate connections instead one changes the synapse model to rate_connection and removes the parameter delay from the synapse dictionary. For the simultaneous use of delayed and instantaneous connections one duplicates lines 17–28 and adapts the synapse and connection dictionaries of the copy according to the needs of the additional instantaneous connections.

**Script 1:** **Simulation of an excitatory-inhibitory network of linear rate units**.

1	**import** nest
2	
3	*# Disable usage of waveform-relaxation method*
4	nest.SetKernelStatus({’resolution’: h, ’use_wfr’: False})
5	
6	*# Create rate units and recording device*
7	n_e = nest.Create(’lin_rate_ipn’, NE,
8	params = {’linear_summation’: True,
9	’mean’: mu, ’std’: sigma, ’tau’: tau})
10	n_i = nest.Create(’lin_rate_ipn’, NI,
11	params = {’linear_summation’: True,
12	’mean’: mu, ’std’: sigma, ’tau’: tau})
13	mm = nest.Create(’multimeter’, params = {’record_from’: [’rate’],
14	’interval’: h, ’start’: T_start})
15	
16	*# Specify synapse and connection dictionaries*
17	syn_e = {’weight’: w, ’delay’: d,
18	’model’: ’delay_rate_connection’}
19	syn_i = {’weight’: -g * w, ’delay’: d,
20	’model’: ’delay_rate_connection’}
21	conn_e = {’rule’: ’fixed_outdegree’, ’outdegree’: KE}
22	conn_i = {’rule’: ’fixed_outdegree’, ’outdegree’: KI}
23	
24	*# Connect rate units*
25	nest.Connect(n_e, n_e, conn_e, syn_e)
26	nest.Connect(n_i, n_i, conn_i, syn_i)
27	nest.Connect(n_e, n_i, conn_i, syn_e)
28	nest.Connect(n_i, n_e, conn_e, syn_i)
29	
30	*# Connect recording device to rate units*
31	nest.Connect(mm, n_e + n_i)
32	
33	*# Start simulation*
34	nest.Simulate(T)

*Here and in the following scripts we use the syntax of the PyNEST interface (Eppler et al.*, *2009**) of the NEST simulation software as of version 2.10.0 (Bos et al.*, *2015**). The script excludes the definitions of the parameters (*h,NE,NI,mu,sigma,tau,T_start,w,d,g,KE,KI,T*)*.

Script 2 shows a code snippet from a simulation script employing model (30) used for mean-field analysis of complex networks in Section 3.3.4. Here single rate units of type siegert_neuron represent an entire population of spiking neurons (lines 3-7). The units are coupled by connections of type diffusion_connection. This connection type is identical to type rate_connection for instantaneous rate connections except for the two parameters drift_factor and diffusion_factor substituting the parameter weight (lines 11–13). These two parameters reflect the prefactors in front of the rate variable in Equations (28) and (29). In general the prefactors differ from these well known forms; for example in case of distributed connection weights (see Helias et al., 2008, their Equation 33). Therefore, we prefer a generic parameterization over more specific alternatives like the pair weight and convergence.

**Script 2:** **Connecting units of typesiegert_neuron**.

1	**import** nest
2	
3	*# Create one siegert_neuron for the excitatory population*
4	s_ex = nest.Create(’siegert_neuron’, 1)
5	*# Create one siegert_neuron for the inhibitory population*
6	s_in = nest.Create(’siegert_neuron’, 1)
7	
8	[… ]
9	
10	*# Create connections originating from the excitatory unit*
11	syn_e = {’drift_factor’: tau_m * K * w,
12	’diffusion_factor’: tau_m * K * w * w,
13	’model’: ’diffusion_connection’}
14	nest.Connect(s_ex, s_ex + s_in, ’all_to_all’, syn_e)
15	
16	[… ]

*The script shows how connections between units of type (30) are created in PyNEST. Again the code snippet does not contain the definitions of the parameters (*tau_m,K,w*) for brevity*.
